# Testing the Effects of dl-Alpha-Tocopherol Supplementation on Oxidative Damage, Total Antioxidant Protection and the Sex-Specific Responses of Reproductive Effort and Lifespan to Dietary Manipulation in Australian Field Crickets (*Teleogryllus commodus*)

**DOI:** 10.3390/antiox4040768

**Published:** 2015-12-04

**Authors:** C. Ruth Archer, Sarah Hempenstall, Nick J. Royle, Colin Selman, Sheridan Willis, James Rapkin, Jon D. Blount, John Hunt

**Affiliations:** 1 Max Planck Research Group, Laboratory of Survival and Longevity, Max Planck Institute for Demographic Research Konrad-Zuse-Str. 1, 18057 Rostock, Germany; E-Mail: C.Archer@exeter.ac.uk; 2 MaxNetAging School, Max Planck Institute for Demographic Research, Konrad-Zuse-Straße 1, 18057 Rostock, Germany; 3 Centre for Ecology and Conservation, College of Life and Environmental Sciences, University of Exeter, Tremough Campus, Cornwall TR10 9FE, UK; E-Mails: N.J.Royle@exeter.ac.uk (N.J.R.); sw360@exeter.ac.uk (S.W.); jr297@exeter.ac.uk (J.R.); J.D.Blount@exeter.ac.uk (J.D.B); 4 Leiden University Medical Center, Postzone S4-P, P.O. Box 9600, 2300 RC Leiden, The Netherlands; E-Mail: S.Hempenstall@lumc.nl; 5 Institute of Biodiversity, Animal Health and Comparative Medicine, College of Medical, Veterinary & Life Sciences, Graham Kerr Building, University of Glasgow, Glasgow G12 8QQ, UK; E-Mail: Colin.Selman@glasgow.ac.uk

**Keywords:** geometric framework of nutrition, free radical theory, reactive oxygen species, sexual selection, vitamin E

## Abstract

The oxidative stress theory predicts that the accumulation of oxidative damage causes aging. More generally, oxidative damage could be a cost of reproduction that reduces survival. Both of these hypotheses have mixed empirical support. To better understand the life-history consequences of oxidative damage, we fed male and female Australian field crickets (*Teleogryllus commodus*) four diets differing in their protein and carbohydrate content, which have sex-specific effects on reproductive effort and lifespan. We supplemented half of these crickets with the vitamin E isoform dl-alpha-tocopherol and measured the effects of nutrient intake on lifespan, reproduction, oxidative damage and antioxidant protection. We found a clear trade-off between reproductive effort and lifespan in females but not in males. In direct contrast to the oxidative stress theory, crickets fed diets that improved their lifespan had high levels of oxidative damage to proteins. Supplementation with dl-alpha-tocopherol did not significantly improve lifespan or reproductive effort. However, males fed diets that increased their reproductive investment experienced high oxidative damage to proteins. While this suggests that male reproductive effort could elevate oxidative damage, this was not associated with reduced male survival. Overall, these results provide little evidence that oxidative damage plays a central role in mediating life-history trade-offs in *T. commodus*.

## 1. Introduction

Aging is the decline in physiological performance over the life-course of an individual, which reduces fertility and increases the risk of mortality. While there is still much to be discovered on the mechanisms that drive aging [1], one theory is that the accumulation of oxidative damage caused by Reactive Oxygen Species (ROS) is responsible [2]. ROS are formed by the incomplete reduction of oxygen, primarily in the mitochondria during aerobic metabolism (discussed in [3]). Because ROS are highly reactive, cells use them as signalling molecules to regulate cellular processes including repair and apoptosis [4]. However, this reactivity also means that ROS can react with biological molecules, such as lipids, proteins and DNA, causing oxidative damage. This damage can impair the function of these molecules, for example, protein oxidation can disrupt enzymatic activity [5]. ROS homeostasis is therefore a compromise between having sufficient ROS to fulfil normal cellular functions and preventing oxidative damage to cells [6]. Antioxidants are vital to this process because they detoxify ROS, rendering them inert [6]. When ROS production exceeds antioxidant defences, cells enter a pro-oxidant state called oxidative stress and damage occurs. If this damage is not repaired then it accumulates in cells [6]. This accumulation of oxidative damage could cause aging [2].

Empirical support for the hypothesis that oxidative damage causes aging is mixed. For example, in keeping with the oxidative stress theory, longer-lived western terrestrial garter snake (*Thamnophis elegans*) ecomorphs have more efficient antioxidant defenses and an improved ability to repair oxidative DNA damage than shorter lived populations [7,8]. Conversely, the longest living rodent species (*Heterocephalus glaber*) has high levels of oxidative damage and poor antioxidant defenses compared to short lived mice [9]. Within species, measures of oxidative damage often increase with age [10], which supports a role for ROS in aging. However, this is not always the case [11] and damage may actually be lower in older individuals [12]. Finally, while some genetic manipulations of antioxidant levels in transgenic mice affect survival, in general genetic manipulations of key antioxidant enzymes show inconsistent effects on lifespan [13]. Overall, it appears that the association between ROS, antioxidants and aging is complex [14].

ROS may play a general role in life-history evolution by mediating trade-offs, such as between reproduction and survival [15,16,17,18]. In many species increased reproductive effort is associated with reduced lifespan (e.g., [19]). However, the mechanistic basis of this “live fast, die young” life-history strategy is generally not well understood [20]. Oxidative damage could be important if reproductive effort elevates ROS production. If this increase in ROS is not mirrored by up-regulated antioxidant defences or the repair of oxidised molecules, then reproduction could accelerate the accumulation of oxidative damage, reducing survival [15]. This could occur if reproductive effort elevates metabolic rate, which in turn, increases mitochondrial ROS production. While reproduction is often associated with large increases in metabolic rate (e.g., [21]), this increased metabolism does not necessarily lead to greater ROS generation [22]. Reproductive effort could also increase oxidative damage if resources typically used to repair or protect against oxidative damage are reallocated towards reproduction. Antioxidant defences are allocated towards reproductive effort in species such as Zebra finches, which use carotenoids to produce pigmented sexual displays [23]. If increasing reproductive effort improves fitness more than investing in a long life, then antioxidants may be allocated towards reproduction rather than cellular defences. In theory, this may accelerate the accumulation of oxidative damage and reduce lifespan [15].

Empirical studies testing whether oxidative damage underpins the trade-off between lifespan and reproduction have also produced mixed results. For example, a recent meta-analysis of females of mammal and bird species found a positive association between reproductive effort (*i.e.*, offspring number) and oxidative damage [18]. However, when comparing oxidative damage in females that bred and those that did not, the reproductive state was associated with reductions in oxidative damage in certain tissues and markers [18]. One potential explanation for this pattern is that antioxidant protection is up-regulated during reproduction in particular tissues in some species [24]. Clearly our understanding of the role ROS play in the trade-off between reproduction and lifespan is incomplete. One reason for this may be that it is not always clear if study species experience a trade-off between lifespan and reproduction in the experimental conditions they are tested [25]. Additionally, it is difficult to experimentally manipulate reproductive effort to determine how this affects oxidative damage and protection [26]. Finally, our existing understanding is based mostly on data collected in females [18]. If oxidative damage mediates the trade-off between reproduction and survival, then ROS homeostasis should differ across the sexes because sexual selection may promote different life-history strategies in males and females [27].

Sexual selection occurs because one sex, usually females, invests more intensely in each offspring than the other sex [28]. Fundamentally, females make a few large eggs, while males produce many small sperm. This means that to improve their reproductive success, females need time and resources to invest heavily in young, while males must attract and fertilize as many females as possible [28]. To achieve this males may adopt a “live fast and die young” life-history strategy, which selects for high early fertility but a short lifespan in males compared to females [27]. Alternatively, if males gain more mates as they age, perhaps because reproductive success relies on a trait that improves over time (e.g., mass, dominance) (e.g., [29]), then sexual selection may favour the evolution of longer lives in males than females [27]. Clearly, sexual selection influences how heavily each sex invests in reproductive effort *versus* longevity. From a mechanistic perspective, this means that sexual selection may affect how each sex allocates antioxidants to reproduction (e.g., carotenoid mediated sexual display) *versus* cellular protection. For example, where males “live fast and die young”, we might expect to see high oxidative damage early in life as a consequence of intense reproductive effort but little investment in antioxidants for defending cells from oxidative damage. Instead, males may allocate antioxidants towards increased expression of sexually selected traits, e.g., bright plumage. Females that require a long time to successfully produce and rear offspring may be more likely to invest heavily in antioxidant defenses to improve their survival. Few studies have explored the role of oxidative stress in mediating the trade-offs between reproductive effort and lifespan in both sexes [18].

Here, we use dietary manipulation to determine the effects of oxidative damage and antioxidant protection on the association between lifespan and reproduction in male and female Australian field crickets, *Teleogryllus commodus*. In the wild, these generalist omnivorous eat foliage, seeds and seedlings and also cannibalize one another [30]. Crickets are an excellent model for examining the link between reproduction, aging and lifespan [31] because reproductive effort can be easily measured in both sexes. In females, reproductive effort can be measured by counting eggs while in males, it can be quantified through measuring how long males spend calling to attract a mate. This energetically expensive behavior is associated with a four-fold increase in metabolic rate [21] and is a primary determinant of how many mates male crickets attract in natural populations [32]. Our ability to easily measure reproductive effort in both sexes means that crickets have been used as a model to explore the effects of nutrition of lifespan and reproduction. Maklakov *et al.* [33] showed that in *T. commodus*, reproduction and lifespan are expressed best in crickets that eat different amounts and blends of key nutrients. Specifically, reproductive effort is highest in females that eat equal amounts of protein and carbohydrate and in males that consume a carbohydrate rich diet [33]. Both sexes, however, live longest when eating low protein, high carbohydrate diets [33]. Sex-specific dietary optima mean that we can use diet to independently manipulate life-history traits in either sex and assay correlated changes in oxidative damage and protection.

We fed crickets four diets that affect lifespan and reproductive effort differently across the sexes [34]. When our stocks of *T. commodus* are fed these diets, no single diet is associated with maximum lifespan and reproductive effort. However, in each sex, one diet is associated with high overall performance, that is, allows very good (but not maximal) survival and reproductive effort [34]. This suggests that diet may mediate a trade-off between these traits in this species, albeit one that is not particularly severe. We supplemented half of these animals with the antioxidant vitamin E isoform dl-alpha-tocopherol and measured nutrient intake, lifespan and reproductive effort (recording time spent calling to attract mate in males—[19], counting eggs in females—[35]) in all crickets. In a subset of individuals, we also measured nutrient intake, as well as assaying total antioxidant capacity (TAC) and oxidative damage to proteins (PC). We regressed values for each of these traits onto protein and carbohydrate intake data to create nutrient landscapes [36] and analyzed these data using powerful response surface methodologies. This is the first application of this method to testing how macronutrients and micronutrients (dl-alpha-tocopherol) interact to affect life-history strategies, oxidative damage and antioxidant protection. We predict that if there is a trade-off between lifespan and reproductive effort in one or both sexes, then these traits should be maximally expressed in animals fed different diets. This is because, if traits are expressed best when crickets are fed different P:C ratios, then crickets cannot express both traits maximally at the same time. We recognise that if two traits share a dietary optimum but animals are fed a restricted diet, then there may be a resource allocation based trade-off between these traits. However, in our experiment where crickets can eat *ad libitum*, trade-offs are clearly signaled when two traits are expressed best in different regions of the nutrient landscape. If oxidative damage explains variation in lifespan following dietary manipulation, then damage should be greatest in animals fed diets that reduce survival. Finally, if oxidative damage is a cost of reproduction that could explain the lifespan-reproduction trade-off, we predict that increased reproductive effort should elevate oxidative damage and/or reduce antioxidant defences. We find mixed and sex-specific support for these hypotheses.

## 2. Materials and Methods

### 2.1. Experimental Animals and Experimental Design

The crickets used in this experiment originate from Smith’s Lake in northern New South Wales, Australia (32°24′ S, 152°28′ E) and were maintained in two 100 L plastic tubs, at a density of over 400 crickets per tub, for five generations prior to this experiment. On the day of hatching, nymphs were individually housed in plastic containers (5 cm × 5 cm × 5 cm) and maintained in a constant temperature room set to 28 °C with a 13:11 L:D cycle. Nymphs were given a piece of cardboard egg carton for shelter and water was provided in a 2.5 mL test tube plugged with cotton wool. Food (Friskies Go-Cat Senior^®^ cat pellets, Nestlé S.A., Vevey, Switzerland) and water were replaced weekly. For the first three feedings, food was ground into powder using a blender and provided in the lid of a 2.5 mL Eppendorf (Fisher Scientific, Waltham, MA, USA). For subsequent feedings, two pellets were provided each week. Once nymphs reached their final developmental instar they were checked daily for eclosion to adulthood.

At eclosion, animals were weighed and randomly allocated to one of two experiments (Experiments 1 and 2). In Experiment 1, we examined the effect of diet and antioxidant supplementation on lifespan and reproduction in males and females. In Experiment 2, we examined the effect of diet and antioxidant supplementation on oxidative damage and antioxidant protection in the sexes. For each experiment, 10 crickets of each sex were randomly allocated to one of eight dietary treatments in a factorial design. These diets varied in their ratio of protein to carbohydrate (P:C ratio of 1:3, 1:1, 3:1 and 5:1) and whether or not they were supplemented with the antioxidant dl-alpha-tocopherol (+AOX and −AOX) to produce the following 8 dietary treatments: (1:3 + AOX, 1:3 − AOX, 1:1 + AOX, 1:1 − AOX, 3:1 + AOX, 3:1 − AOX, 5:1 + AOX and 5:1 − AOX). From here-on, we refer to animals fed DL-alpha-tocopherol as “supplemented” and those that did not receive dl-alpha-tocopherol as non-supplemented. Our total sample size was 320 crickets but some escaped or died due to a handling death, so our final sample size was 312 crickets.

### 2.2. Construction of Artificial Diets

Crickets were fed one of four, dry granular diets (Figure S1, Table S1) that differed in the ratio of protein to carbohydrate (P:C) and were made according to methods detailed in Simpson and Abisgold [37] and applied in Maklakov *et al.* [33] to *T. commodus*. Each of these four diets were either supplemented with bound dl-alpha tocopherol (a synthetic, stable form of vitamin E) at 12 mg per 100 g of diet or left non-supplemented. The food given to stock cricket populations (Friskies Go Cat Senior) contains this antioxidant and so we felt confident it would not have pathological effects on experimental animals. Additionally, vitamin E may be important in insect reproduction, but its exact role is unclear [38]. We chose a level of supplementation equal to the concentration of vitamin E in Friskies Go Cat Senior, which the crickets have evolved under for five generations. An assay of dl-alpha-tocopherol levels in each of our animals in Experiment 2 using high-performance liquid chromatography (HPLC) showed that supplemented crickets had significantly higher levels of dl-alpha-tocopherol than non-supplemented crickets, thereby confirming that our supplementation regime was successful (Text S1, Figures S2–S4). To add this antioxidant to the diets, bound dl-alpha tocopherol was dissolved in 2 mL of ethanol and vortexed for five minutes at 15 hz in a glass vial. This solution was dissolved in chloroform and introduced to the diets alongside the other water insoluble ingredients. For diets that were not supplemented with dl-alpha-tocopherol, 2 mL of ethanol was added to the chloroform as a control. Each diet was stored under inert N_2_ gas at −80 °C and new batches of diet were made each month of the experiment. Diets were dried, which removed all solvent, prior to feeding.

### 2.3. Feeding Protocol

Adult crickets were kept in plastic containers (17 × 12 × 6 cm) with a 6 cm long piece of plastic tubing (2 cm diameter) for shelter. Water was provided in the upturned plastic lid of a vial (1.6 cm diameter, 1.6 cm deep) glued to a plastic petri-dish (5.5 cm in diameter). Food was provided in a slightly larger feeding platform of the same design (vial lid: 2.1 cm diameter, 1.2 cm deep petri-dish). The materials used for housing meant that experimental animals could not eat anything other than their diets and petri-dish bases collected any food spilt from feeding bowls. Food of known dry mass was provided on the day of eclosion. Three days later, this food was removed and replaced with new food of known mass. Food was replaced every three or four days (see below). When diet was retrieved from animals, faeces were removed and diets were dried at 30 °C for 72 h prior to weighing. Consumption was calculated as the difference in dry weight before and after feeding and these values were converted to a weight of protein and carbohydrate consumed. Crickets were checked for mortality and water daily.

We mated experimental animals weekly throughout the experiment to ensure that female fecundity was not restricted by sperm limitation. We also mated males because mating can affect lifespan and we wished to ensure that survival data were comparable across the sexes. We first mated animals on day six post eclosion when food was removed and a randomly chosen stock animal of the opposite sex introduced to the container. This mate was removed 24 h later (*i.e.*, day 7), when new food was provided. This procedure was repeated weekly (*i.e.*, at days 6, 13, 20, *etc*.) until animals died (Experiment 1) or were killed (Experiment 2). In Experiment 2, crickets were re-weighed and euthanized by freezing at −80 °C. Crickets were killed at day 17 because this is just below the mean lifespan seen for females fed a high protein diet [34] and therefore should allow time for physiological differences between animals on different diets to accumulate but not select for particularly long lived crickets. Where crickets did not survive to day 17, they were replaced with a new individual.

### 2.4. Experiment 1: The Effect of Diet and dl-Alpha-Tocopherol Supplementation on Lifespan and Reproductive Effort

Reproductive effort was measured on days 3 and 7 post eclosion and once a week thereafter (*i.e.*, day 14, 21, 28 post eclosion, *etc.*) throughout the cricket’s life (Experiment 1) or until day 17 (Experiment 2). To measure female reproductive effort, females were provided with a petri-dish (5 cm diameter) full of moist sand in which to oviposit. Females had oviposition substrate throughout the experiment, except when paired with mates. Egg dishes were removed when females were given a new mate and replaced when the mate was removed. The contents of each petri-dish was emptied into a plastic, circular container (10 cm diameter, 10 cm height), 200 mL of water added and the container swirled in a circular motion for 10 s. This caused any eggs to move to the surface of the sand, where they were removed with fine forceps and counted. In males, we used calling effort (the time each male spent calling on a given night) as our measure of reproductive effort. In this species, time spent calling is a good measure of reproductive effort because calling is energetically expensive [21] and females strongly prefer males that call more in both the laboratory [39] and the field [32]. We recorded the calling effort of each male using a custom-built electronic monitoring device that sampled each male 10 times per second from 6 P.M. to 9 A.M. Full details of this device are provided in Hunt *et al*. [19]. All crickets were checked daily for survival.

### 2.5. Experiment 2: The Effect of Diet and dl-Alpha-Tocopherol Supplementation on Oxidative Damage and Antioxidant Protection

All biochemical assays were completed within three months of freezing at −80 °C. Animals were thawed and their gut and crop removed to ensure that only antioxidants and proteins that had been assimilated into tissues were assayed and not those contained within undigested food. Post dissection, crickets were re-weighed and then immediately homogenized for thirty seconds in 2 mL of phosphate buffered saline (pH 7.4) in falcon tubes using an Ultra-Turrax T-28 homogenizer, (IKA, Staufen, Germany). Homogenate was centrifuged at 13,000 rpm for 20 min at 4 °C in 2 mL Eppendorfs. The supernatant fraction was separated and centrifuged for a further 5 min. This final supernatant was divided into aliquots for each assay and stored in 1.5 mL Eppendorfs at −80 °C.

Protein concentration was determined using the Bradford method [40] using a spectrophotometer (Spectramax Plus384, Molecular Devices, Sunnyvale, CA, USA). This quantifies the milligrams of protein in 1 mL of cricket homogenate. We assayed protein carbonyl group formation as a measure of oxidative damage. Protein carbonyls (PCs) are stable moieties produced by most types of ROS on oxidation of proteins [5]. Concentration of protein carbonyls was assayed using a commercially available kit (Protein Carbonyl Colorimetric Assay Kit, Cayman Chemical, Ann Arbor, MI, USA, Item no. 10005020). In short, the PC assay relies upon the reaction between protein carbonyl groups and 2,4-dinitrophenylhydrazine (DNPH). This reaction produces a stable product that may be quantified spectrophotometrically at an absorbance between 360 and 385 nm. Samples were diluted five-fold with homogenization buffer and assayed in duplicate. Values from this assay are presented in nmol/mL but presented here as nmol/mg protein.

Total antioxidant capacity (TAC) was also measured using a commercially available kit (Antioxidant Assay Kit, Cayman Chemical, Ann Arbor, MI, USA, Item no. 709001). These kits measure the oxidation of ABTS (2,2′-Azino-di-[3-ethybenzthiazoline sulphonate]) by metmyoglobin. This oxidative reaction is inhibited by antioxidants, and so by comparing the degree of inhibition to a standard curve constructed using known amounts of the inhibitor (Trolox), the total antioxidant capacity of sample can be ascertained. Samples were run in duplicate and diluted 100-fold. Values are quantified as millimolar Trolox equivalents. dl-alpha-tocopherol was assayed using the methods in Supplementary Information Text S1.

### 2.6. Statistical Analyses

To estimate the linear and nonlinear effects of P and C intake on our response variables (lifespan, reproductive effort, PC and TAC) in the sexes, we used a multivariate response-surface approach. In brief, this method first fits a model that quantifies how expression of a trait changes as intake of each nutrient increases linearly. Non-linear terms are then built into this model. First, a correlational effect is included that tests how intake of each nutrient interacts to affect trait expression. Finally, a quadratic effect of each nutrient is included, which measures curvature in the data. If adding each of these terms sequentially improves the fit of the model, then it indicates a significant linear, correlational or quadratic effect of each nutrient on trait expression.

As per convention [41], response surfaces for each response variable were run hierarchically. That is, a model was first run that only included the linear terms for nutrient intake (P and C) and then a second model was run that included all linear and nonlinear terms for nutrient intake (P, C, P × P, C × C and P × C). The linear coefficients (*i.e.*, estimates of the linear effects of P and C on trait values) were taken from this first model and the nonlinear coefficients from the second complete model. This second model only included linear terms to ensure that the nonlinear coefficients provided an estimate of the curvature of the response surface that was free from directionality [41]. As the total intake of nutrients and reproductive effort depends on lifespan, which is known to vary across the sexes in *T. commodus* [33], we expressed values in milligrams per day. Furthermore, because females are larger than males in *T. commodus* and this influences the daily intake of nutrients, we also expressed the daily intake of nutrients per milligram of cricket. Similarly, levels of PC and TAC were expressed per mg protein to control for the fact that larger crickets contain greater levels of protein. Thus, our final values of PC and TAC are presented per mg/mL of protein, P and C intake were measured in mg eaten per day/per mg of cricket, whereas our measures of reproductive effort (egg production and calling effort) were measured per day. As our measures of nutrient intake and our response variables were measured in different units, we standardized each measure to a mean of zero and standard deviation of one using a Z-transformation prior to analysis to facilitate the direct statistical comparison of response surfaces and to ensure that any observed differences are not simply the result of differences in scale. We fitted response surfaces in JMP (version 8.0.2, SAS Institute, Cary, NC, USA) and we visualized them using “TPS” function implemented in the “fields” package [42] of R (R Core Team, version 2.13.0 ,Vienna, Austria). For each surface, we used the value of the smoothing parameter (λ) that minimized the generalized cross-validation (GCV) score [43]. Although we conducted all analyses on standardized data, we present raw data in our thin-plate splines to ease interpretation. We interpret these response surfaces as nutritional landscapes.

Both nutrient intake and our response variables deviated significantly from a normal distribution. Thus, although we extracted linear and nonlinear regression coefficients for nutritional landscapes using multiple regression, we tested their significance using randomization tests implemented in Poptools (version 3.2, Canberra, Australia). We started by running a response surface model that included only the linear terms. Next we randomly shuffled the response variable across diets to obtain a null distribution under random association and the same response surface model run on the response variable. A Monte-Carlo simulation was used to repeat this 10000 times. The number of permutations (out of 9999) in which the linear coefficient for the randomly shuffled data was greater than or equal to the linear coefficient from the structured data was calculated and this proportion (*p*) converted to a two-tailed probability value for each term in the model as 2 *p* if *p* < 0.5 or as 2 (1 − *p*) if *p* > 0.5 [44]. This procedure was repeated for the complete response surface model for each response variable to provide a significance test for the nonlinear coefficients.

We used a sequential model-building approach to determine whether the linear and nonlinear effects of P and C intake on our different response variables differed with DL-alpha-tocopherol supplementation and across the sexes [45]. We also used this approach to test whether the linear and nonlinear effects of nutrients differed across our response variables within each of these levels. Full details and an application of this approach to nutritional data are provided in the online supplement (Text S2). In brief, this approach sequentially adds the linear (P and C), quadratic (P × P and C × C) and correlational (P × C) effects of nutrients as covariates to a general linear model including a fixed effect containing two or more levels (e.g., dl-alpha-tocopherol supplementation, sex or the type of response variable). At each stage of this sequence, the amount of variance unexplained by a reduced model containing the covariates and fixed effect is compared to the variance unexplained by a more complete model that also contains all interactions between the fixed effect and the covariates using a partial *F*-test that accounts for the increased terms added to the complete model and change in degrees of freedom [46,47]. If the addition of interaction terms improves the fit of the complete model (*i.e.*, the unexplained variance is reduced), this provides evidence that the covariates have different effects on the response variable across the fixed effect. Inspection of the individual interaction terms from the complete model can be used to determine exactly which covariate(s) is driving this effect.

While this sequential model-building approach provides a direct statistical test for differences in the magnitude of linear, quadratic and correlational effects on the response variables across the fixed effect being compared, it does not provide information on the direction of this difference in nutritional space. It is therefore possible that the response variables show differences in the magnitude of any effects but are optimized in similar regions on the nutritional landscape. We therefore also calculated the angle (***θ***) between the linear vectors for the two response variables being compared using trigonometry and the 95% confidence intervals for ***θ*** using a Bayesian approach. When ***θ*** = 0° the vectors are perfectly aligned and the optima for the two response variables reside in the same location in nutrient space, whereas ***θ*** = 180° represents the maximum possible divergence between vectors. Full details of these calculations and accompanying R code are provided in the online supplement (Text S3).

## 3. Results

### 3.1. The Effect of Diet and dl-Alpha-Tocopherol Supplementation on Lifespan and Reproductive Effort

**Figure 1:**
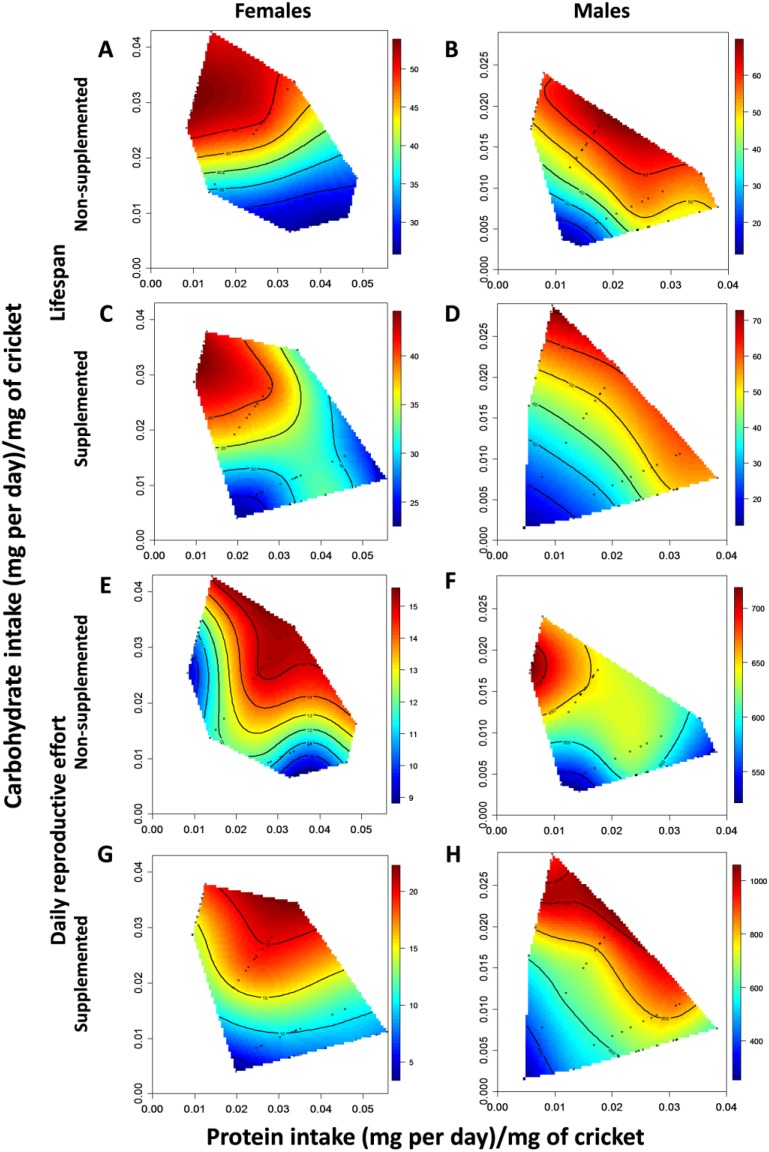
Nutritional landscapes illustrating the effects of daily protein and carbohydrate intake on lifespan (**A**–**D**) and daily reproductive effort (**E**–**H**) in female (**A**, **C**, **E**, **G**) and male (**B**, **D**, **F**, **H**) crickets that were supplemented with DL-alpha-tocopherol (**C**, **D**, **G**, **H**) or not (**A**, **B**, **E**, **F**). High values of these traits are in red and low values in blue. The scale associated with each graph is shown to its right: the values for each scale bar are days (for lifespan), daily number of eggs laid (female reproductive effort) or time spent calling (male reproductive effort). Black dots represent the actual intake of nutrients by individual crickets.

In both supplemented and non-supplemented females, lifespan increased with carbohydrate intake, peaking at a daily intake of approximately 0.032 mg of C per day/mg of cricket and a P:C ratio of 1:3 (Figure 1A,C, Table 1 (A)). In supplemented females, there was also a significant negative correlational coefficient showing that lifespan was maximized in females that consumed a high carbohydrate, low protein diet (Figure 1C, Table 1 (A)). Formal comparison however, showed that the effects of protein and carbohydrate intake on lifespan did not differ significantly between supplemented and non-supplemented females (Table S2 (A)). Finally, a small angle (33.99°) between linear vectors for lifespan indicates that survival peaks in the same region of nutritional space for supplemented and non-supplemented females (Figure 1A,C, Table S2 (A)).

In contrast to lifespan, daily reproductive effort was influenced by intake of protein and carbohydrate (Table 1 (A)). Irrespective of supplementation status, female fecundity increased with carbohydrate intake and peaked at an intermediate intake of protein (~ 0.03 mg of P per day/mg of cricket) (Figure 1E,G, Table 1 (A)). There was a significant difference in the linear (but not quadratic or correlational) effects of nutrient intake on daily fecundity across supplemented and non-supplemented females (Table S2 (A)). This difference arose because fecundity was more sensitive to carbohydrate intake (*i.e.*, increased more steeply with carbohydrate consumption) in supplemented females than non-supplemented females (Table S2 (A)). Accordingly, fecundity peaked at approximately 20 eggs per day in supplemented females and around 15 eggs per day in non-supplemented females (Figure 1E,G). However, the small angle (34.34°) between linear vectors shows that fecundity peaked in similar regions of the nutritional landscape for supplemented and non-supplemented females (Figure 1E,G, Table S2 (A)).

There was a significant difference in the linear effects of nutrients on lifespan and reproduction in supplemented and non-supplemented females. In both cases this was driven by carbohydrate (Table S2 (A)). In supplemented females, fecundity was more sensitive to carbohydrate intake than lifespan was, whereas the opposite was true for non-supplemented females (Table 1 (A)). In addition to this difference in sensitivity to carbohydrate intake, the large angle between linear vectors (51.30° and 54.59° for supplemented and non-supplemented females, respectively) indicates that the peaks for trait expression fell in different areas of the nutrient landscape. Daily fecundity peaked around a P:C ratio of 1:1 (Figure 1E,G), whereas the peaks for lifespan occurred around a 1:3 P:C ratio (Figure 1A,C). Consequently, the P:C ratio that maximizes fecundity reduces lifespan (and *vice versa*) leading to an inevitable trade-off between these traits.

In both supplemented and non-supplemented males, lifespan increased significantly with carbohydrate intake and, to a lesser degree, protein intake (Figure 1B,D, Table 1 (B)). The linear, quadratic and correlational effects of nutrients on male lifespan did not differ between supplemented and non-supplemented males (Table S2 (B)) and lifespan was maximized at around 60 days in both cases. Furthermore, the smaller angle (27.14°) between linear vectors demonstrates that lifespan peaked in a similar region of the nutritional landscape (Figure 1B,D, Table S2 (B)). Male calling effort was influenced by carbohydrate intake but not by protein (Figure 1F,H, Table 1 (B)). In supplemented males, calling effort increased linearly with carbohydrate intake (Figure 1H, Table 1 (B)), whereas in non-supplemented males, calling effort peaked just below the maximum intake of carbohydrate (~ 0.018 mg of C per day/mg of cricket, Figure 1F, Table 1 (B)). Despite this, the linear, quadratic and correlational effects of nutrients on calling effort did not differ significantly across supplemented and non-supplemented males. Additionally, the small angle (35.96°) between linear vectors demonstrates that calling effort was maximized in similar regions of the nutritional landscape (Figure 1F,H, Table S2 (B)).

**Table 1:** The linear and nonlinear effects of daily protein (P) and carbohydrate (C) intake on the lifespan (LS) and daily reproductive effort (DRE) of (**A**) female and (**B**) male *T. commodus* with and without dl-alpha-tocopherol supplementation. Significant effects are highlighted in bold.

	Non-Supplemented			Supplemented		
	Coefficient * ± SE	Prop	*p* Value	Coefficient ± SE	Prop	*p* Value
**(A): Females**						
*LS*						
P	−0.159 ± 0.171	0.181	0.362	−0.095 ± 0.183	0.695	0.610
C	**0.486 ± 0.159**	**0.998**	**0.004**	**0.271 ± 0.126**	**0.019**	**0.038**
P × P	−0.258 ± 0.198	0.102	0.203	−0.350 ± 0.201	0.953	0.094
C × C	**−0.374 ± 0.176**	**0.021**	**0.042**	**−0.307 ± 0.144**	**0.018**	**0.039**
P × C	−0.283 ± 0.266	0.853	0.295	**−0.661 ± 0.276**	**0.012**	**0.023**
*DRE*						
P	0.192 ± 0.165	0.125	0.250	0.139 ± 0.196	0.758	0.484
C	**0.280 ± 0.133**	**0.022**	**0.042**	**0.678 ± 0.168**	**0.001**	**0.002**
P × P	**−0.187 ± 0.083**	**0.016**	**0.031**	**−0.416 ± 0.193**	**0.018**	**0.037**
C × C	−0.145 ± 0.126	0.129	0.259	−0.204 ± 0.194	0.851	0.298
P × C	0.179 ± 0.262	0.751	0.498	−0.260 ± 0.291	0.810	0.379
**(B): Males**						
*LS*						
P	**0.521 ± 0.202**	**0.994**	**0.013**	**0.511 ± 0.193**	**0.995**	**0.010**
C	**0.961 ± 0.302**	**0.002**	**0.003**	**0.767 ± 0.227**	**0.001**	**0.002**
P × P	−0.422 ± 0.290	0.912	0.176	−0.137 ± 0.277	0.689	0.622
C × C	−0.635 ± 0.480	0.905	0.191	−0.224 ± 0.322	0.245	0.490
P × C	−0.379 ± 0.468	0.785	0.430	−0.292 ± 0.453	0.737	0.526
*DRE*						
P	−0.023 ± 0.257	0.536	0.928	0.170 ± 0.276	0.729	0.543
C	0.086 ± 0.317	0.605	0.790	**0.354 ± 0.149**	**0.021**	**0.043**
P × P	−0.126 ± 0.308	0.659	0.683	−0.201 ± 0.334	0.274	0.549
C × C	**−0.935 ± 0.421**	**0.017**	**0.033**	−0.064 ± 0.388	0.565	0.870
P × C	−0.858 ± 0.676	0.106	0.212	−0.060 ± 0.547	0.543	0.914

* The linear regression coefficients (*i.e.*, P and C) describe the linear slope of the relationship between nutrient intake and the response variable, whereas the quadratic regression coefficients (*i.e.*, P × P and C × C) describes the curvature of this relationship, with a negative coefficient indicating a convex relationship (*i.e.*, a peak on the response surface) and a positive coefficient indicating a concave relationship (*i.e.*, a trough on the response surface). The correlational regression coefficients (*i.e.*, P × C) describe how the covariance between the two nutrients influences the response variable, with a negative coefficient indicating that a negative covariance between nutrients increases the response variable and a positive coefficient indicating that a positive covariance between nutrients increases the response variable. Full details of this approach are provided in Lande and Arnold [41]. “*p* value” is the significance value and “prop” is the proportion of times out of 10,000 that the shuffled gradient exceeds the normal gradient, for discussion please see the Methods section.

There were significant differences in the linear effects of nutrients on male lifespan and reproductive effort (Table S2 (B)). Irrespective of supplementation status, male lifespan was more responsive to carbohydrate intake than calling effort was. In supplemented males, both traits were maximized at a P:C ratio of 1:3 and a small angle (40.14°) separated the linear vectors for these traits (Figure 1D,H, Table S2 (B)). In non-supplemented males, lifespan and calling effort were maximized at a P:C ratio of 1:1 and 1:3 respectively and the angle between the linear vectors was larger (53.60°), indicating that these traits are maximized in different regions of the nutritional landscape (Figure 1B,F, Table S2 (B)). While this provides some evidence for a trade-off between reproduction and lifespan, it is clearly less pronounced than in females as it only appears in non-supplemented males.

Across the sexes, there was a significant difference in the linear effects of nutrients on lifespan in both supplemented and non-supplemented crickets (Table S3). In supplemented crickets this was because first, male lifespan was positively affected by protein intake while female lifespan was not. Second, male lifespan was more sensitive to carbohydrate intake than female lifespan. In non-supplemented crickets differences for lifespan across the sexes arose because male lifespan increased with protein intake but female lifespan did not. The angles between the linear vectors for lifespan were large, at 72.35° and 68.07° for supplemented and non-supplemented crickets respectively. For daily reproductive effort, only the linear effects of nutrient intake in supplemented animals differed across the sexes. This was because female fecundity was more sensitive to carbohydrate intake than male calling effort (Table S3). However, the angles between linear vectors for daily reproductive effort across the sexes were large at 55.40° (supplemented crickets) and 72.00° (non-supplemented crickets). Together, these findings suggest that irrespective of supplementation status, maximum lifespan and daily reproductive effort occur in different regions of the nutritional landscape for male and female crickets (Figure 1).

### 3.2. The Effect of Diet and dl-Alpha-Tocopherol Supplementation on Oxidative Damage and Antioxidant Protection

**Figure 2:**
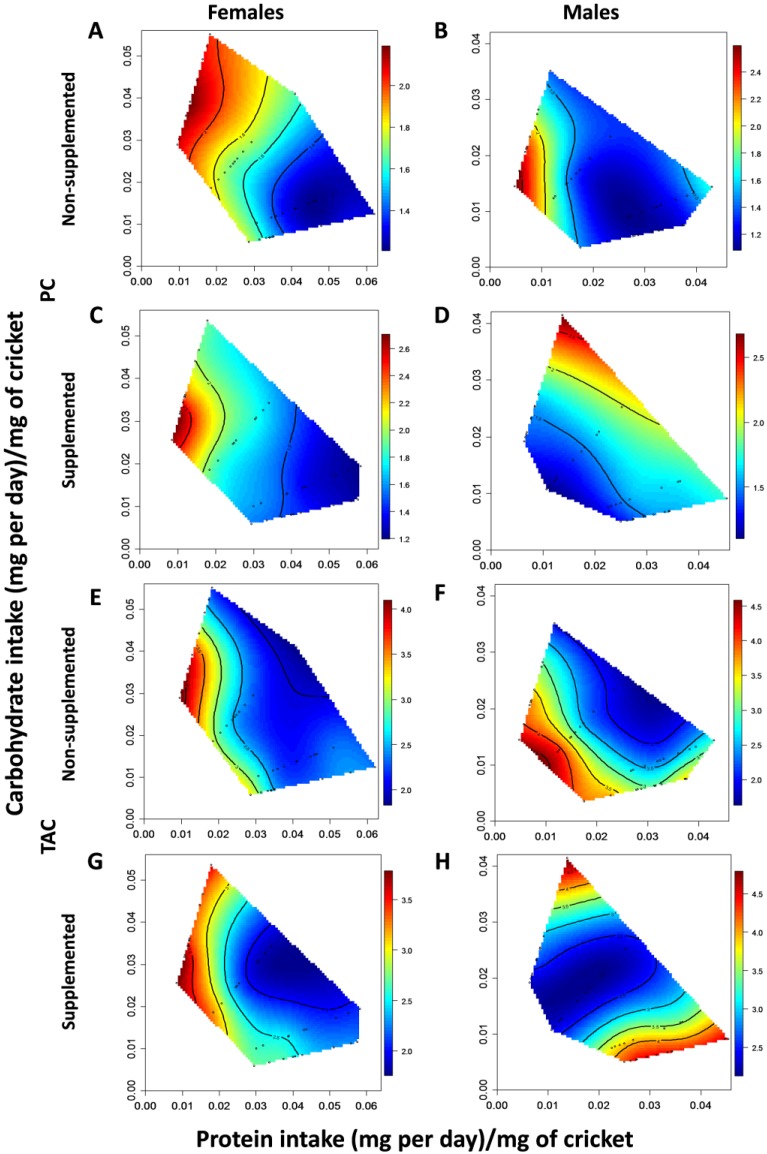
Nutritional landscapes illustrating the effects of daily protein and carbohydrate intake on oxidative damage to proteins (PC, **A**–**D**) antioxidant capacity (TAC, **E**–**H**) in female (**A**, **C**, **E**, **G**) and male (**B**, **D**, **F**, **H**) crickets that were supplemented with DL-alpha-tocopherol (**C**, **D**, **G**, **H**) or not (**A**, **B**, **E**, **F**). The scale associated with each graph is shown to its right: the values for each scale bar are presented per mg of protein in cricket homogenate (PC: nmol, TAC: mmol). High values of these traits are in red and low values in blue. Black dots represent the actual intake of nutrients by individual crickets.

In both supplemented and non-supplemented females, oxidative damage (measured as protein carbonylation) decreased significantly with increasing protein intake (Figure 2A,C, Table 2 (A)). A similar pattern was observed for antioxidant protection; TAC levels also decreased with protein intake (Figure 2E,G, Table 2 (A)). Peaks for these traits occurred in similar regions of the nutritional landscape, irrespective of whether females were supplemented with additional antioxidants or not. This is illustrated by similar linear, quadratic and correlational effects of nutrient intake on PC and TAC in supplemented and non-supplemented females (Table S4A) and the small angles separating the linear vectors for these traits (PC AOX+ *vs*. AOX−: 32.00°; TAC AOX+ *vs*. AOX−: 41.64°) (Figure 2A,C,E,G, Table S4 (A)). Comparing diet effects on these different traits within supplemented and non-supplemented females, the effects of nutrient intake on levels of PC and TAC did not significantly differ and the angles between linear vectors were small (37.49° and 38.41° for supplemented and non-supplemented females, respectively). This suggests that in females, PC levels peaked in the same region of the nutritional landscape as levels of TAC (Figure 2A,C,E,G, Table S4 (A)).

**Table 2:** The linear and nonlinear effects of daily protein (P) and carbohydrate (C) intake on the protein carbonylation (PC) and total antioxidant capacity (TAC) of (**A**) female and (**B**) male *T. commodus* with and without dl-alpha-tocopherol supplementation. Significant effects are highlighted in bold.

	Non-Supplemented			Supplemented		
	Coefficient ± SE	Prop	*p* Value	Coefficient ± SE	Prop	*p* Value
**(A): Females**						
*PC*						
P	**−0.347 ± 0.162**	**0.981**	**0.039**	**−0.300 ± 0.143**	**0.022**	**0.043**
C	0.099 ± 0.157	0.266	0.532	0.026 ± 0.188	0.445	0.890
P × P	0.272 ± 0.217	0.891	0.218	0.084 ± 0.227	0.643	0.714
C × C	0.160 ± 0.147	0.858	0.284	−0.092 ± 0.210	0.105	0.211
P × C	0.307 ± 0.330	0.179	0.358	−0.074 ± 0.426	0.568	0.864
*TAC*						
P	**−0.249 ± 0.119**	**0.978**	**0.043**	**−0.217 ± 0.091**	**0.011**	**0.022**
C	−0.076 ± 0.170	0.329	0.657	−0.042 ± 0.196	0.415	0.830
P × P	0.347 ± 0.231	0.928	0.144	0.199 ± 0.234	0.799	0.401
C × C	0.062 ± 0.156	0.347	0.694	0.138 ± 0.217	0.735	0.530
P × C	0.228 ± 0.352	0.739	0.521	0.057 ± 0.439	0.449	0.898
**(B): Males**						
*PC*						
P	**−0.355 ± 0.169**	**0.022**	**0.044**	**0.439 ± 0.212**	**0.023**	**0.046**
C	0.065 ± 0.299	0.586	0.827	**0.584 ± 0.279**	**0.022**	**0.043**
P × P	**0.969 ± 0.280**	**0.001**	**0.002**	0.012 ± 0.379	0.512	0.976
C × C	0.057 ± 0.364	0.562	0.876	0.258 ± 0.322	0.215	0.429
P × C	0.600 ± 0.573	0.151	0.303	0.016 ± 0.709	0.509	0.981
*TAC*						
P	**−0.406 ± 0.197**	**0.977**	**0.047**	0.300 ± 0.265	0.868	0.265
C	−0.424 ± 0.304	0.087	0.173	0.032 ± 0.287	0.456	0.912
P × P	**0.478 ± 0.230**	**0.023**	**0.046**	0.092 ± 0.355	0.399	0.797
C × C	0.023 ± 0.428	0.521	0.958	**0.392 ± 0.145**	**0.005**	**0.011**
P × C	−0.161 ± 0.674	0.594	0.811	−0.778 ± 0.664	0.875	0.250

In non-supplemented males, levels of oxidative damage decreased with protein intake, reaching a minimum at approximately 0.025 mg P per day/mg of cricket (Figure 2B, Table 2 (B)). In contrast, in supplemented males oxidative damage increased with protein and carbohydrate intake (Figure 2D, Table 2 (B)). Additionally, the linear and quadratic effects of nutrient intake on levels of PC differed between supplemented and non-supplemented males (Table S4 (B)). The linear difference resulted from protein intake having a positive effect on levels of PC in supplemented males (*i.e.*, increased damage) but a negative effect in non-supplemented males (*i.e.*, reduced damage). The quadratic difference resulted from a significant minimum for protein intake in non-supplemented males but not for supplemented males (Table 2 (B)). Accordingly, the angle between the linear vectors was large (90.95°) indicating that levels of PC were maximized in different regions of the nutritional landscape for supplemented and non-supplemented males (Figure 2B,D, Table S4 (B)).

Antioxidant defenses in non-supplemented males decreased significantly with protein intake and TAC levels reached a minimum at approximately 0.03 mg protein per day/mg of cricket (Figure 1F, Table 2 (B)). In contrast, levels of TAC in supplemented males were minimized at an intermediate intake of carbohydrate (approximately 0.02 mg per day/mg of cricket—Figure 2H, Table 2 (B)). The linear, quadratic and correlational effects of nutrients on TAC expression did not differ between supplemented and non-supplemented males (Table S4 (B)). Despite this, the angle between linear vectors was large (105.90°) indicating that levels of TAC were maximized in different regions of the nutritional landscape (Figure 2F,H, Table S4 (B)). As shown for females, the linear, quadratic and correlational effects of nutrition on levels of PC and TAC did not differ significantly within supplemented or non-supplemented males (Table S4 (B)). However, unlike in females the angles between linear vectors were large being 90.23° and 67.63° for supplemented and non-supplemented males, respectively. This indicates that levels of PC and TAC peaked in different regions of the nutritional landscape (Figure 2B,D,F,H, Table S4 (B)).

Across the sexes, there was a significant difference in the linear effects of nutrients on levels of PC in supplemented crickets (Table S5). This difference was due to the fact that PC levels decreased with protein intake in supplemented females but increased with protein intake in supplemented males. In addition, a large angle between the linear vectors (94.09°) show that peak levels of PC occurred in different regions of the nutritional landscape in either sex (Figure 2C,D, Table S5). There was also a significant difference in the quadratic (but not linear or correlational) effects of nutrients on levels of PC across the sexes in non-supplemented crickets due to the fact that there was a significant minimum for protein intake in non-supplemented males but not in non-supplemented females (Table S5). The small angle (33.65°) between linear vectors shows that minimum levels of PC in male and female non-supplemented crickets occupy a similar region on the nutritional landscape (Figure 2A,B, Table S5). In contrast to levels of PC, no significant differences in the linear, quadratic or correlational effects of nutrients on levels of TAC across the sexes were detected in either supplemented or non-supplemented crickets (Table S5). However, the angle between linear vectors for supplemented crickets was large (107.80°) indicating that TAC levels peaked in different regions of the nutritional landscape in supplemented males and females (Figure 2G,H, Table S5). In contrast, this angle (40.43°) was much smaller in non-supplemented crickets and maximum levels of TAC occupied a similar region of the nutritional landscape (Figure 2E,F, Table S5).

### 3.3. Do Differences in Oxidative Damage or Protection Mediate Life-History Trade-Offs?

In non-supplemented females, the effects of nutrients on lifespan and levels of PC did not differ significantly and the angle between the linear vectors for these traits was small (37.24°). This indicates that survival and oxidative damage peaked in similar regions of the nutritional landscape (Figure 1A and Figure 2A, Table S6). In contrast, there was a significant difference in the linear effects of nutrition on lifespan and levels of TAC in non-supplemented females. This reflects that lifespan increased with carbohydrate intake but TAC levels did not (Table S6). The angle between the linear vectors for these traits was large (81.20°) indicating that they were maximized in different regions of the nutritional landscape and therefore are likely to trade-off (Figure 1A and Figure 2E, Table S6). There were also significantly different linear effects of nutrients on fecundity and levels of PC in non-supplemented females (Table S6). This difference reflects that levels of PC decreased with protein intake while fecundity did not. This led to a large angle (76.39°) between the linear vectors for these traits, illustrating that they are maximized at different regions of the nutritional landscape (Figure 1E and Figure 2A, Table S6). In contrast, the linear, quadratic and correlational effects of nutrients on fecundity and levels of TAC did not differ significantly in non-supplemented females (Table S6). Despite this, there was a large angle (94.70°) between the linear vectors for fecundity and TAC indicating that these traits are maximized at different regions of the nutritional landscape (Figure 1E and Figure 2E, Table S6).

In supplemented females, the linear, quadratic and correlational effects of nutrients on lifespan and levels of PC and on lifespan and levels of TAC did not differ significantly (Table S6). Furthermore, the angles between these traits were small (38.71° and 43.56°, respectively) indicating that the maxima for these trait values were located in similar regions of the nutritional landscape (Figure 1C and Figure 2C,G, Table S6). In contrast, there were significant differences in the linear and quadratic (but not correctional) effects of nutrients on fecundity and levels of PC and on fecundity and levels of TAC in supplemented females (Table S6). The difference in linear effects occurred because fecundity was more sensitive to the intake of carbohydrate than levels of both PC and TAC. The differences in the quadratic effects occurred because there was a peak in fecundity with protein intake but not in levels of PC and TAC (Table S6). The angles between the linear vectors for these traits were large, being 72.35° between fecundity and PC and 86.24° between fecundity and TAC, indicating that these traits are maximized in different regions of the nutritional landscape and are therefore more likely to trade-off (Figure 1G and Figure 2C,G, Table S6).

In non-supplemented males, the linear and quadratic effects of nutrients on lifespan and levels of PC differed (Table S7). The difference in linear effects was due to lifespan increasing with the intake of both nutrients, whereas levels of PC decreased with protein intake and was not influenced by carbohydrate (Table S7). This resulted in lifespan and levels of PC being maximized at different regions of the nutritional landscape, as evidenced by the large (73.10°) angle between the linear vectors for these traits (Figure 1B and Figure 2B, Table S7). The difference in quadratic effects arose because levels of PC reached a significant minimum at an intermediate protein intake but lifespan did not (Table S7). There was also a significant difference in the linear (but not quadratic or correlational) effects of nutrients on lifespan and levels of TAC. This reflects that lifespan increased with the intake of both nutrients, whereas levels of TAC decreased with protein intake and was not influenced by carbohydrate intake (Table S7). A large (116.00°) angle between the linear vectors for lifespan and TAC shows that these traits are maximized at different regions in nutritional space (Figure 1B and Figure 2F, Table S7). In contrast, there were no significant differences in the linear, quadratic or correlational effects of nutrients on calling effort and levels of PC, as well as on calling effort and levels of TAC (Table S7). However, in both instances the angles between linear vectors were large, being 79.57° and 104.60° respectively, indicating these traits were maximized at different regions of the nutritional landscape (Figure 1F and Figure 2B,F, Table S7).

Finally, in supplemented males the linear, quadratic and correlational effects of nutrients on lifespan and levels of PC did not differ significantly and there was a small angle (25.52°) between the linear vectors for these traits. This indicates that they are maximized in similar regions of the nutritional landscape (Figure 1D and Figure 2D, Table S7). In contrast, there was a significant difference in the linear (but not quadratic or correlational) effects of nutrients on lifespan and levels of TAC. This results from the fact that lifespan increased with carbohydrate intake but levels of TAC did not (Table S7). A large angle (92.18°) between the linear vectors for these traits show that they are maximized in different regions of the nutritional landscape (Figure 1D and Figure 2H, Table S7). The linear, quadratic and correlational effects of nutrients on calling effort and levels of PC and on calling effort and levels of TAC did not significantly differ (Table S7). For PC levels and calling effort, the angle between linear vectors was small (42.52°) indicating the maxima for these traits occurs in similar regions on the landscape (Figure 1H and Figure 2D, Table S7). For TAC and calling effort, this angle was much larger (103.50°) suggesting that these traits peak in different regions on the nutritional landscape (Figure 1H and Figure 2H, Table S7).

## 4. Discussion

Although the oxidative stress theory is a widely accepted mechanistic theory of aging, it has mixed empirical support [14,48]. It is also unclear if oxidative damage mediates the trade-off between lifespan and reproduction that is observed in many species [15,18,49]. To understand the life-history effects of oxidative damage, we used diet to manipulate lifespan and reproductive effort in male and female Australian field crickets. We then examined how this affected oxidative damage to proteins and antioxidant protection. Although we found that female crickets face a trade-off between lifespan and reproductive effort, oxidative damage to proteins did not appear to mediate this trade-off. In males fed dl-alpha-tocopherol, daily reproductive effort elevated oxidative damage. This suggests that oxidative damage could be a cost of reproduction. However, this did not appear to reduce male survival. Clearly, the life-history consequences of oxidative damage are sex-specific and complex (Table 3).

**Table 3:** A summary of the main conclusions drawn by this study.

Hypothesis	Prediction	Observation	Conclusion	
Trade-off between lifespan and reproduction.	Traits peak in different regions of nutrient landscape.	Females—prediction met. Males—prediction met in non-supplemented males but not in supplemented males.	Sex-specific trade-off, pronounced in females, weak in males.	
Accumulation of oxidative damage causes aging.	Damage highest in short lived animals.	Generally, high damage, long lifespan.	Oxidative damage to proteins does not appear to mediate variation in lifespan within each sex, following dietary manipulation.	
	Antioxidant supplementation improves lifespan.	No effect of antioxidants on survival.		
Reproductive effort elevates oxidative stress.	High reproductive effort reduces antioxidant defences.	Mixed support: results depend on sex and supplementation status.	Association between oxidative damage and reproductive effort varies enormously across the sexes.	
	High reproductive effort increases oxidative damage.			
	Antioxidant supplementation improves reproductive effort.	Weak positive effect but not significant.		

### 4.1. The Effect of Nutrient Intake on Life-History Traits

In both sexes, protein (P) and carbohydrate (C) had different effects on lifespan and reproductive effort. Female lifespan peaked in individuals that ate a 1:3 P:C ratio, while fecundity was optimized on a 1:1 P:C ratio. This suggests that females cannot eat a single diet to simultaneously maximize their reproductive output and their survival and so illustrates a trade-off between these traits. In males, lifespan was greatest in animals that consumed large amounts of protein and carbohydrate, while calling effort was primarily influenced by carbohydrate intake. Peak calling effort was best aligned with intake of the 1:1 P:C diet, while lifespan was greatest in crickets that consumed large amounts of the 1:1 P:C or 1:3 P:C diet. Formal comparison of nutrient landscapes for these traits, showed a trade-off between lifespan and reproductive effort in non-supplemented males but not in supplemented males. Clearly, males do not face a pronounced trade-off between these traits.

Our results differ slightly from previous work on *T. commodus*. While we found that protein intake had a positive effect on male survival, Maklakov and colleagues [33] found that the protein consumption reduced male lifespan. This difference may reflect that our experimental animals were mated, while in Maklakov *et al.* [33] males were virgins. Mating might increase a male’s requirement for protein to make sperm [50] or to improve immune function [51] after mating, which can promote the spread of disease [52]. This differences in male and female requirements for protein meant that nutrient landscapes for lifespan differed across the sexes: males required more protein and less carbohydrate than females to maximise their survival. Nutrient landscapes for reproduction also differed significantly across the sexes, which is not surprising given that males and females rely on very different traits (calling effort *versus* egg laying) to secure reproductive success.

### 4.2. Testing the Oxidative Stress Theory of Aging

If the oxidative stress theory holds true, then we can predict that dl-alpha-tocopherol supplementation should increase lifespan. Alternatively, diets that reduce survival should elevate oxidative damage. We did not find support for either prediction. dl-alpha-tocopherol did not affect survival and diets that improved survival were generally associated with elevated oxidative damage. In females, lifespan, oxidative damage and antioxidant protection peaked in medium to high carbohydrate, low protein regions of the nutrient landscape. Although these traits differed in how strongly they responded to protein or carbohydrate intake, nutritional landscapes for these traits did not differ significantly. Similarly in supplemented males, diets that promoted a long life were associated with high oxidative damage. The only exception to this trend was in non-supplemented males where high levels of oxidative damage did not overlap with diets that were particularly good (or particularly bad) for survival.

We cannot completely dismiss a role for oxidative damage in mediating differences in lifespan within each sex. Levels of oxidative damage often differ between tissue types and depend on the measure of damage that is assayed [18,26]. In theory, diets which increased lifespan in *T. commodus* may have reduced oxidative damage to tissue types that we did not measure. However, life-extending dietary manipulations do not consistently reduce oxidative damage across species and tissues (see Table S8). This makes it seem unlikely that reduced oxidative damage is the only mechanism by which diets improve lifespan.

Instead, we found that high protein intake was associated with poor survival and low levels of oxidative damage in non-supplemented males and in all females. High protein intake is detrimental to survival in many species [53] and this has often been attributed to elevated oxidative damage [54,55]. Our results largely contradict this theory. One explanation for our results may be that consuming protein allows better turnover of oxidized proteins [56]. The synthesis of protein itself can reduce lifespan [57] and the currently unidentified costs of protein synthesis may be a mechanism by which protein intake reduces lifespan independently of oxidative stress. More research is needed to identify the physiological costs of a high protein intake and protein synthesis [57].

### 4.3. Testing a Role for Oxidative Damage in Life-History Trade-Offs

If oxidative damage is a cost of reproduction, we would expect that dl-alpha-tocopherol supplementation would increase reproductive effort and/or diets that promote intense reproductive effort would be associated with high oxidative damage or low antioxidant protection. We find mixed support for these hypotheses. In females, dl-alpha-tocopherol did not significantly improve fecundity. However, females fed diets high in carbohydrate laid 33% more eggs per day when they were supplemented with dl-alpha-tocopherol than when they were not. This weak and non-significant difference raises the possibility that dl-alpha-tocopherol may interact with carbohydrate intake to improve female fecundity. In many invertebrate species, dl-alpha-tocopherol is involved in female reproduction and can improve egg production [38,58,59]. Additionally, synergistic interactions between antioxidants and different nutrients are widespread [60]. For example, in African honeybees (*Apis melliera scutellata*) supplementation with the main antioxidant in green tea, epigallocatechin-3-gallate, only improved survival in bees fed low amino acid, high carbohydrate diets [61]. However, more research is needed to confirm this hypothesis. In males, dl-alpha-tocopherol did not significantly affect calling effort but had a weak positive effect on how long males called each night. These very slight differences suggest that oxidative damage may be a constraint on reproductive effort and that supplementary antioxidants may improve reproductive effort. However, it is clear that these effects are very weak in *T. commodus*. Perhaps had we used the more potent RRR alpha-tocopherol isoform [62], or a higher supplementation dose, supplementation may have significantly improved trait expression but this requires further investigation.

A stronger test for a role of oxidative damage in mediating life-history trade-offs is in seeing how reproductive effort affects oxidative damage and antioxidant protection. In females, diets that improved fecundity did not increase oxidative damage. This suggests that oxidative damage to proteins does not underpin the trade-off between reproductive effort and lifespan in female *T. commodus.* Similarly, in non-supplemented males, protein carbonyls and calling effort peaked in different regions of the nutritional landscape. However, in supplemented males, calling effort and protein carbonylation fell in a similar area of the nutritional landscape. This indicates that diets associated with reproductive effort in supplemented males were also associated with high protein oxidative damage. We cannot say if this represents a causal relationship but it is easy to imagine that it might. In *T. commodus* calling effort is associated with a four-fold increase in metabolic rate [21]. This might increase ROS leakage from the mitochondria and accelerate the accumulation of damage. Although, the association between metabolic rate and oxidation is complex and not always positive [22,63], in some cricket species high metabolic rate is associated with reduced lifespan [64], suggesting that elevated metabolism carries some physiological costs. That there is only an overlap between calling effort and protein damage in supplemented males but not non-supplemented males might reflect the qualitative but non-significant increase in calling effort seen in males fed DL-alpha-tocopherol. Clearly this result is speculative, and even where reproductive effort was associated with high oxidative damage this did not lead to large reductions in lifespan.

Finally, our data show that peaks for oxidative damage and antioxidant protection largely overlap in females, but less so in males. This may suggest that when ROS production increases, as indicated by higher protein oxidation, stored antioxidants are mobilized or antioxidant production is up-regulated in females. However, it appears that this up-regulation is insufficient to prevent oxidative damage accumulating. Males do not show such signs of active up-regulation of antioxidant protection. It seems reasonable to suggest that males cannot up-regulate their antioxidant capacity. This may be because they have insufficient resources, or because they are allocating resources to an alternative trait. Overall, this result illustrates that the association between oxidative damage, antioxidant protection and life-history traits is complex and shaped by sex-specific selection pressures.

## 5. Conclusions

Our results show that oxidative damage to proteins does not explain variation in lifespan within male and female *T. commodus* following dietary manipulation. This adds to a large body of evidence showing that while ROS may be involved in aging and in mediating lifespan, there are clearly other mechanisms involved. We also find that the association between oxidative damage and reproduction is sex-specific. dl-alpha-tocopherol has a weak positive effect on reproductive effort in both sexes, but supplementation did not result in significantly higher daily reproductive effort. Elevated reproductive effort was not closely associated with high oxidative damage in females. However, the diets that favoured elevated male calling effort were associated with high oxidative damage but only in males fed dl-alpha-tocopherol. This suggests that calling may increase oxidative damage by elevating ROS production or by reducing the ability of cells to repair oxidative damage, rather than by reducing antioxidant levels.

We suggest that the approach we adopt here—using fine-scale dietary manipulation to test the mechanistic basis of life-history trade-offs—is potentially a powerful one. Despite results being complicated, they show that the association between reproductive effort and oxidative stress is sex-specific and depends on how reproductive effort is measured [18]. However, it is increasingly clear that to really understand how ROS affect life-histories, we must measure different biomarkers of damage, repair and antioxidant activities, in a range of tissue types. This is because levels of oxidative damage and antioxidant activity often vary between different biomarkers and tissues. This means that the conclusions drawn by studies such as ours depend enormously on how oxidative damage and protection are measured [26]. In summary, our results highlights the importance of studying both sexes in testing the life-history consequences of ROS and illustrates that unravelling the mechanisms underlying dietary effects on life-history trade-offs may prove to be a very difficult task.

## Supplementary Files

Supplementary File 1
