# Supplementary material for: Testing the Effects of dl-Alpha-Tocopherol Supplementation on Oxidative Damage, Total Antioxidant Protection and the Sex-Specific Responses of Reproductive Effort and Lifespan to Dietary Manipulation in Australian Field Crickets (Teleogryllus commodus)
Source: Antioxidants (Basel). 2015 Dec 4;4(4):768–92. doi: 10.3390/antiox4040768 (PMC4712936; doi:10.3390/antiox4040768)
Supplement: Supplementary File 1 [file antioxidants-04-00768-s001.pdf]

Supplementary Materials: Testing the Effects of DL-Alpha-Tocopherol Supplementation on Oxidative Damage, Total Antioxidant Protection and the Sex-Specific Responses of Reproductive Effort and Lifespan to Dietary Manipulation in Australian Field Crickets (*Teleogryllus commodus*)

C. Ruth Archer, Sarah Hempenstall, Nick J. Royle, Colin Selman, Sheridan Willis, James Rapkin, Jon D. Blount and John Hunt

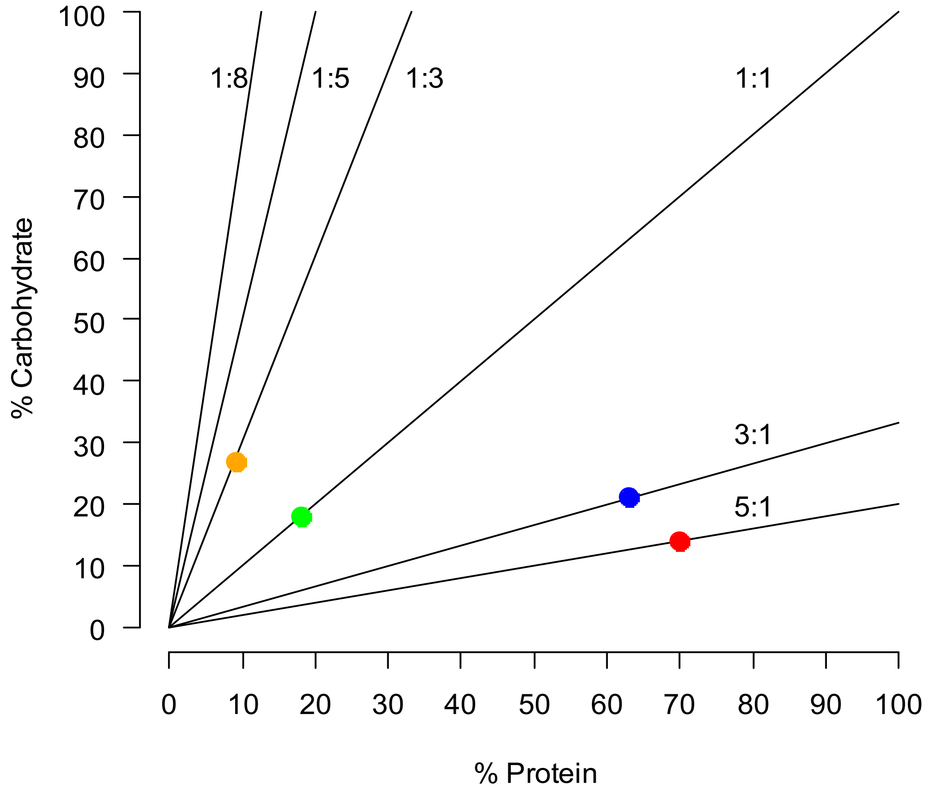

Figure S1. The nutrient content of the diets used in this experiment. Points represent the protein and carbohydrate composition of the four diets used. The ratio of protein to carbohydrate within each diet is represented by black lines or "nutrient rails". We plot all the nutrient rails used in an earlier study of *T. commodus* [1] to allow comparison. In this study [1] diets were numbered, 4 (red), 8 (blue), 10 (green) and 14 (yellow).

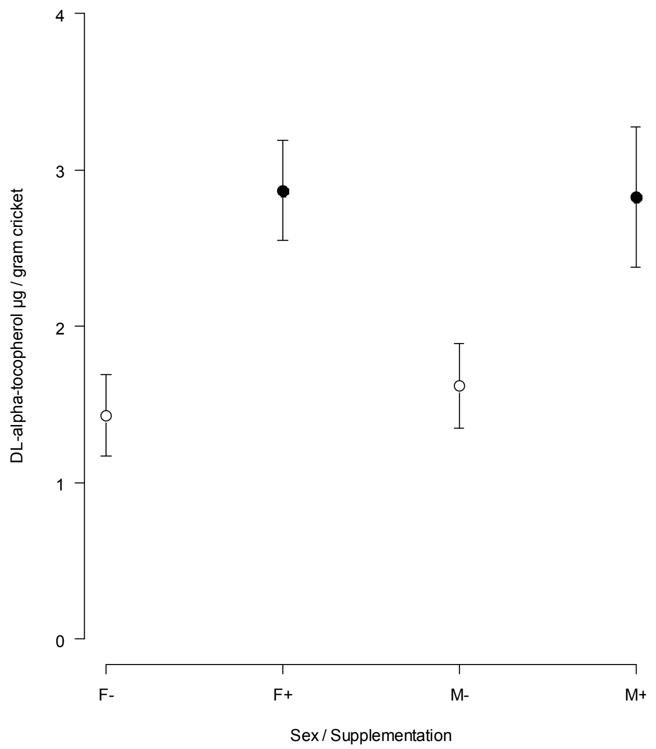

Figure S2. Mean DL-alpha-tocopherol levels in females (F) and males (M) that were supplemented (+, closed circles) or not (−, open circles) with DL-alpha-tocopherol. Error bars represent standard errors.

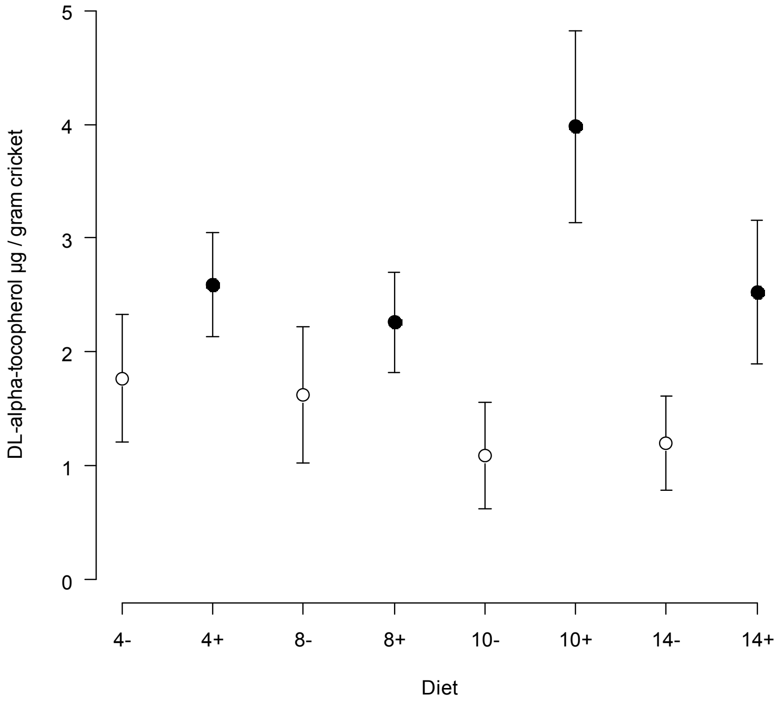

Figure S3. The DL-alpha-tocopherol content of female crickets fed each of the diets used in this experiment (4, 8, 10, 14) and supplemented (+, closed circles) or not (−, open circles) with DL-alpha-tocopherol. Diets are as in Figure S1, error bars are standard errors around the mean.

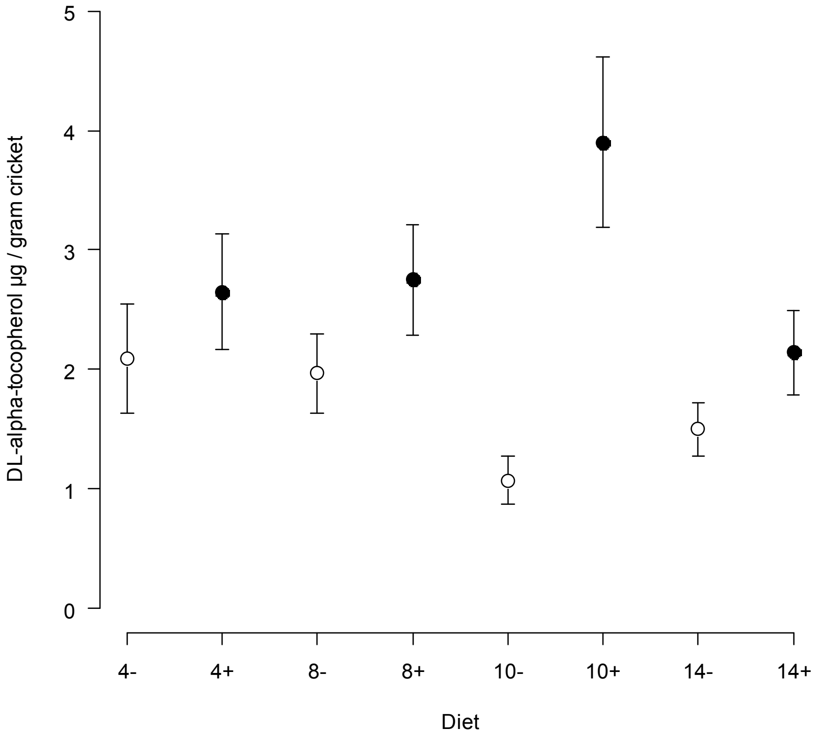

Figure S4. The DL-alpha-tocopherol content of male crickets fed each of the diets used in this experiment (4, 8, 10, 14) and supplemented (+, closed circles) or not (−, open circles) with DL-alpha-tocopherol. Diets are as in Figure S1, error bars are standard errors around the mean.

Text S1. Confirming that Our DL-Alpha-Tocopherol Supplementation Treatment Was Successful

To measure DL-alpha-tocopherol, 5 mL of pyrogallol was added to 300 µL of whole cricket homogenate in a 40 mL glass vial. Pyrogallol prevented any oxidation of DL-alpha-tocopherol. 1 mL of KOH was added to each sample for saponification. Samples were recapped under N_2 gas and placed in a water bath at 70 °C for 30 min. 5 mL of hexane was then added to extracted DL-alpha-tocopherol. 10 mL of Milli-Q was added to the samples, all samples were recapped under N_2 gas and put on a bench-top mixing plate for five minutes. They were then centrifuged at 10,000 RPM at 4 °C for 4 min. 3 mL of the hexane layer was extracted and dried down in an evaporating rotary chamber for 20 min. Once completely evaporated, 100 uL of 0.05% butylated hydroxytouene (BHT) in ethanol was added to dissolve the DL-alpha-tocopherol. Samples were then vortexed for 30 s at 15 Hz. 100 uL of sample was injected into a Dionex HPLC system fitted with a Waters Spherisorb 3 um ODS2 column (4.6 × 150 mm, Hertfordshire, UK). The mobile phase was methanol to water (97:3), run isoratically for 15 min. DL-alpha-tocopherol was detected using a fluorescence detector, with an excitation wavelength of 330 nm and an emission wavelength of 480 nm. Peak area was quantified using a standard curve prepared from alpha-tocopherol standard. The peaks allowed for quantitative amounts of µg of vitamin per gram of cricket to be calculated.

To test if DL-alpha-tocopherol levels were higher in supplemented animals, we used the "glm" function in R. Sex, diet and supplementation level were included as explanatory variables, as were all the interactions between them, and DL-alpha-tocopherol content per mg of cricket was our response variable. DL-alpha-tocopherol levels were log transformed prior to analyses. Significance of terms was

assessed via backwards model simplification and non-significant terms removed when $P < 0.05$. Model simplification showed that neither sex ($F_{149,148} = 0.083$, $P = 0.773$), diet ($F_{150,149} = 0.940$, $P = 0.334$) or the interaction between the two ($F_{147,146} = 0.654$, $P = 0.654$) significantly affected the DL-alpha-tocopherol content of samples. The only variable that significantly influenced the content of DL-alpha-tocopherol in tissue homogenate was the supplementation status of crickets ($F_{151,150} = 25.892$, $P = < 0.001$) *i.e.*, if they were fed DL-alpha-tocopherol or not.

Text S2. Sequential Model Building Approach to Compare Nutritional Landscapes for Lifespan, Reproductive Effort, Oxidative Damage and Antioxidant Protection

We used a sequential model building approach to determine if the linear and nonlinear effects of protein and carbohydrate consumption were different across our response variables [2,3]. Because our response variables (e.g., lifespan *versus* female fecundity) were measured in different scales (e.g., days *versus* eggs laid), we standardized them for statistical comparison to make sure that any differences we see in the linear or nonlinear effects of nutrient intake are not driven simply by differences in scale. To do this, we used a *Z*-transformation to standardize each response variable and nutrient intake to a mean of zero and standard deviation of one. We then included a dummy variable, response type (*RT*), in a reduced model containing only the standardized linear terms:

$$R = \beta_0 + \alpha_0 RT + \sum_{i=1}^{n} \beta_i N_i + \varepsilon \quad (1)$$

where R is our standardized response variables, N_i refers to the intake of the ith nutrient, n represents the number of nutrients contained in the model and ε is the unexplained error.

From Equation (1), the unexplained (*i.e.*, residual) sums of squares for this reduced model (SS_r) was compared to the same quantity (SS_c) from a second (complete) model that included all of the terms in Equation (1) with the addition of the terms $\alpha_i N_i RT$ which represents the linear interaction of RT and the ith nutrient.

$$R = \beta_0 + \alpha_0 RT + \sum_{i=1}^{n} \beta_i N_i + \sum_{i=1}^{n} \alpha_i N_i RT + \varepsilon \quad (2)$$

To compare SS_r and SS_c from (Equation (1)) and (Equation (2)) respectively we used a partial F-test [4]:

$$F_{a,b} = \frac{(SS_r - SS_c)/a}{SS_c/b} \quad (3)$$

where a is the number of terms that differ between the reduced and complete model while b is the error degrees of freedom for SS_c.

To test whether the quadratic effect of nutrient intake differed across response variables, the SS_r from the reduced model:

$$R = \beta_0 + \alpha_0 RT + \sum_{i=1}^{n} \beta_i N_i + \sum_{i=1}^{n} \alpha_i N_i RT + \sum_{i=1}^{n} \beta_i N_i^2 + \varepsilon \quad (4)$$

was compared to the SS_c of the complete model:

$$R = \beta_0 + \alpha_0 RT + \sum_{i=1}^{n} \beta_i N_i + \sum_{i=1}^{n} \alpha_i N_i RT + \sum_{i=1}^{n} \beta_i N_i^2 + \sum_{i=1}^{n} \alpha_i N_i^2 RT + \varepsilon \quad (5)$$

using (Equation (3)).

To test if the correlational effects of nutrient intake on response variables differed, the SS_r from the reduced model:

$$R = \beta_0 + \alpha_0 RT + \sum_{i=1}^{n} \beta_i N_i + \sum_{i=1}^{n} \alpha_i N_i RT + \sum_{i=1}^{n} \beta_i N_i^2 + \sum_{i=1}^{n} \alpha_i N_i^2 RT + \sum_{i=1}^{n} \sum_{j\geq 1}^{n} \beta_{ij} N_i N_j + \varepsilon \quad (6)$$

was compared to the SS_c of the complete model:

$$R = \beta_0 + \alpha_0 RT + \sum_{i=1}^{n} \beta_i N_i + \sum_{i=1}^{n} \alpha_i N_i RT + \sum_{i=1}^{n} \beta_i N_i^2 + \sum_{i=1}^{n} \alpha_i N_i^2 RT + \sum_{i=1}^{n} \sum_{j\geq 1}^{n} \beta_{ij} N_i N_j + \sum_{i=1}^{n} \sum_{j\geq 1}^{n} \alpha_{ij} N_i N_j RT + \varepsilon \quad (7)$$

using (Equation (3)).

This approach means that the comparison of model (Equation (1)) *versus* (Equation (2)), (Equation (4)) *versus* (Equation (5)), and (Equation (6)) *versus* (Equation (7)) tests for the overall significance of the interaction between response type and the linear, quadratic and correlational effects of nutrient intake, respectively. Significant differences in these model comparisons (identified with a partial F-test) therefore show that the linear, quadratic and/or correlational effects of nutrient intake on the response variables differ. Finally, we also considered the interaction of different nutrients with the response variable terms from the full model (Equation (7)) to determine if intake of protein, carbohydrate or both nutrients were responsible for the significance of the overall partial F-test.

Text S3. The Annotated R Code That Was Used to Estimate the Angle (θ), and 95% CIs, between Linear Vectors for the Effects of Nutrients on Lifespan, Reproductive Effort, Oxidative Damage and Antioxidant Protection. The package MCMCglmm, developed by Jarrod Hadfield (https://cran.r-project.org/web/packages/MCMCglmm/index.html) is required to run this code.

```
# load the library
library (MCMCglmm)
# read in nutritional data for first female trait (e.g., offspring number)
angle.data1 < -read.table (“offspring.txt”, h = T)
attach (angle.data1)
str (angle.data1)
# str (angle.data) should give 3 columns for data structure (e.g., offspring number, P intake and C intake)
# Bayesian linear regression to estimate beta for each variable, produces posterior distribution based on 15200 estimates of each parameter:
angle.model.offspring < -MCMCglmm (offspring ~ P + C-1, data = angle.data1, v = 0.02, nitt = 400,000, burnin = 20,000, thin = 25)
summary (angle.model.offspring)
```

```
# and again for second female trait (e.g., lifespan):
library (MCMCglmm)
angle.data2 < -read.table (“lifespan.txt”, h = T)
attach (angle.data2)
str (angle.data2)
angle.model.lifespan < -MCMCglmm (lifespan ~ P + C-1, data = angle.data2, v = 0.02, nitt = 400,000, burnin = 20,000, thin = 25)
summary (angle.model.lifespan)
angles < -numeric (15200)
# creates an empty vector the same length as the posterior distribution, in which angle estimates for each row of the posterior distribution will be stored as follows:
for(i in 1:15200){
b.offspring<- angle.model.offspring$Sol[i,1:2]
b. lifespan <- angle.model.lifespan$Sol[i,1:2]
# creates a vector of beta estimates for each variable for each row of the posterior distribution (and the loop runs through all rows)
angles[i]<- acos((t(b. lifespan) %*% b. lifespan)/((sqrt(t(b. lifespan) %*% b. lifespan)) * (sqrt(t(b.offspring) %*% b.offspring)))) * (180/pi)}
# calculates the angles between offspring number and lifespan beta's for each row of the posterior distribution
summary (angles)
# to examine angle estimates which are now stored in the vector called “angles”
# provides the mean, median, minimum and maximum angle. The 1st and 3rd Quantiles are functionally equivalent to the 95% CIs. We use the median and 95% CIs in our manuscript for theta
```

Table S1. The nutrient content of the diets used in this experiment. The total nutrient content of each diet is given as the sum of the percentage protein (P) and percentage carbohydrate (C) (*i.e.*, P + C = 36), the remaining percentage consists of indigestible carbohydrate. Diet numbers allow comparisons to be drawn to an earlier experiment [1] and colors correspond to Figure S1. All diets contained Wesson’s salts (2.5%), ascorbic acid (0.275%), cholesterol (0.55%) and vitamin mix (0.18%). Vitamin mix contains thiamine (1.4%), riboflavin (1.4%), nicotinic acid (5.6%), pyridoxine (1.4%), folic acid (1.4%), Meso-inositol (14%), calcium pantothenate (2.8%), p-aminobenzoic acid (1.4%), Choline chloride (70.4%) and biotin (0.06%).

Protein (P)	Carbohydrate (C)	P + C	P:C	Diet Number
70	14	84	5:1	**4**
63	21	84	3:1	**8**
18	18	36	1:1	**10**
9	27	36	1:3	**14**

Table S2. Sequential model comparing the linear and nonlinear effects of protein (P) and carbohydrate (C) intake on lifespan (LS) and daily reproductive effort (DRE) in (A) female and (B) male *T. commodus* with and without DL-alpha-tocopherol. SS refers to sums of squares of the reduced (SS_r) and the complete (SS_c) models. DF quantifies the degrees of freedom, F the test statistic and θ the angle separating peaks for the traits being compared, with associated 95% confidence intervals.

	SS_R	SS_C	DF_1	DF_2	F	p Value	θ (95% CI)
**(A): Females**							
Supplemented vs. non-supplemented LS							
Linear	56.20	54.58	2	73	1.08	0.34	33.99° (16.05°, 56.89°)
Quadratic	52.30	52.23	2	69	0.05	0.95	
Correlational	47.74	47.06	1	67	0.97	0.33	
Supplemented vs. non-supplemented DRE							
Linear	61.93	56.40	2	73	3.58	**0.03** [A]	34.34° (18.14°, 68.90°)
Quadratic	50.75	50.34	2	69	0.28	0.76	
Correlational	50.28	49.36	1	67	1.28	0.26	
Supplemented LS vs. supplemented DRE							
Linear	70.17	64.27	2	74	3.40	**0.04** [B]	51.30° (23.86°, 74.77°)
Quadratic	63.17	61.03	2	70	1.23	0.30	
Correlational	56.70	55.88	1	68	1.00	0.32	
Non-supplemented LS vs. non-supplemented DRE							
Linear	49.87	44.21	2	72	4.61	**0.01** [C]	54.59° (25.99°, 75.48°)
Quadratic	42.26	41.54	2	68	0.59	0.56	
Correlational	41.49	40.55	1	66	1.53	0.22	
**(B): Males**							
Supplemented vs. non-supplemented LS							
Linear	58.39	58.06	2	71	0.20	0.82	27.14° (12.69°,48.21°)
Quadratic	57.21	55.67	2	67	0.93	0.40	
Correlational	54.31	53.86	1	65	0.54	0.46	
Supplemented vs. non-supplemented DRE							
Linear	73.53	73.10	2	71	0.21	0.81	35.96° (20.09°, 56.20°)
Quadratic	72.46	71.45	2	67	0.47	0.62	
Correlational	70.89	70.06	1	65	0.77	0.38	
Supplemented LS vs. supplemented DRE							
Linear	80.53	74.28	2	71	3.26	**0.04** [D]	40.14° (18.88°, 91.34°)
Quadratic	72.01	71.19	2	67	0.39	0.68	
Correlational	70.92	70.80	1	65	0.11	0.74	
Non-supplemented LS vs. non-supplemented DRE							
Linear	64.72	57.43	2	71	3.00	**0.04** [E]	53.60° (22.12°, 97.71°)
Quadratic	56.47	55.93	2	67	0.32	0.72	
Correlational	53.12	53.11	1	65	0.01	0.92	

Univariate tests: [A] P: $F_{1,73} = 0.21$, $P = 0.84$, C: $F_{1,73} = 5.01$, $P = 0.029$; [B] P: $F_{1,74} = 0.87$, $P = 0.39$, C: $F_{1,74} = 4.85$, $P = 0.031$; [C] P: $F_{1,72} = 0.94$, $P = 0.35$; C: $F_{1,72} = 3.99$, $P = 0.05$; [D] P: $F_{1,71} = 0.90$, $P = 0.35$; C: $F_{1,71} = 3.98$, $P = 0.05$; [E] P: $F_{1,71} = 2.34$, $P = 0.13$; C: $F_{1,71} = 4.08$, $P = 0.047$.

Table S3. Sequential model comparing the linear and nonlinear effects of protein (P) and carbohydrate (C) intake on lifespan (LS) and daily reproductive effort (DRE) across the sexes in *T. commodus* with and without DL-alpha-tocopherol supplementation. SS refers to sums of squares of the reduced (SS_r) and the complete (SS_c) models. DF quantifies the degrees of freedom and F is the test statistic.

	SS_R	SS_C	DF_1	DF_2	F	p Value
Supplemented LS						
Linear	68.80	61.87	2	72	4.03	**0.02** [A]
Quadratic	61.75	60.15	2	68	0.90	0.41
Correlational	55.73	55.31	1	66	0.50	0.48
Non-supplemented LS						
Linear	55.52	50.77	2	72	3.37	**0.04** [B]
Quadratic	48.31	47.75	2	68	0.40	0.67
Correlational	46.15	45.61	1	66	0.78	0.38
Supplemented DRE						
Linear	84.56	77.36	2	72	3.35	**0.04** [C]
Quadratic	72.27	72.07	2	68	0.09	0.91
Correlational	71.49	71.37	1	66	0.11	0.74
Non-supplemented DRE						
Linear	52.25	51.87	2	72	0.26	0.77
Quadratic	50.78	49.71	2	68	0.73	0.48
Correlational	49.71	48.06	1	66	2.27	0.14

Univariate tests: [A] P: $F_{1,72} = 4.20$, $P = 0.044$; C: $F_{1,72} = 3.98$, $P = 0.049$; [B] P: $F_{1,72} = 5.29$, $P = 0.024$; C: $F_{1,72} = 2.02$, $P = 0.16$; [C] P: $F_{1,72} = 0.01$, $P = 0.93$; C: $F_{1,72} = 4.02$; $P = 0.049$.

Table S4. Sequential model comparing the linear and nonlinear effects of protein (P) and carbohydrate (C) on protein carbonylation (PC) and total antioxidant capacity (TAC) in (A) female and (B) male *T. commodus* with and without DL-alpha-tocopherol. SS refers to sums of squares of the reduced (SS_r) and the complete (SS_c) models. DF quantifies the degrees of freedom, F the test statistic and θ the angle separating peaks for the traits being compared, with associated 95% confidence intervals.

	SS_R	SS_C	DF_1	DF_2	F	p Value	θ (95% CI)
**(A): Females**							
Supplemented vs. non-supplemented PC							
Linear	64.66	64.38	2	72	0.16	0.86	32.00° (14.89°, 55.99°)
Quadratic	63.11	62.54	2	68	0.31	0.73	
Correlational	62.21	61.74	1	66	0.50	0.48	
Supplemented vs. non-supplemented TAC							
Linear	72.72	72.70	2	72	0.01	0.99	41.64° (22.52°, 67.60°)
Quadratic	68.94	68.24	2	68	0.35	0.71	
Correlational	67.89	67.79	1	66	0.10	0.76	
Supplemented PC vs. supplemented TAC							
Linear	70.10	69.67	2	72	0.22	0.80	37.49°(22.44°, 82.38°)
Quadratic	67.61	66.66	2	68	0.49	0.62	
Correlational	66.65	66.61	1	66	0.02	0.98	
Non-supplemented PC vs. non-supplemented TAC							
Linear	68.93	67.42	2	72	0.81	0.45	38.41° (23.89°, 79.51°)
Quadratic	64.74	64.12	2	68	0.33	0.72	
Correlational	62.95	62.92	1	66	0.02	0.98	
**(B): Males**							
Supplemented vs. non-supplemented PC							
Linear	72.03	67.14	2	72	3.35	**0.04** ^{A}	90.95° (56.76°, 126.20°)
Quadratic	63.51	55.66	2	68	4.65	**0.01** ^{B}	
Correlational	55.20	54.79	1	66	0.48	0.49	
Supplemented vs. non-supplemented TAC							
Linear	74.54	70.49	2	72	2.07	0.13	105.90° (70.46°, 145.30°)
Quadratic	64.91	61.88	2	68	1.66	0.20	
Correlational	61.02	60.63	1	66	0.42	0.52	
Supplemented PC vs. supplemented TAC							
Linear	72.13	69.27	2	72	1.49	0.23	90.23° (52.01°, 128.40°)
Quadratic	63.37	62.33	2	68	0.57	0.57	
Correlational	61.76	61.14	1	66	0.69	0.41	
Non-supplemented PC vs. non-supplemented TAC							
Linear	70.40	68.71	2	72	0.89	0.42	67.63° (36.10°, 103.30°)
Quadratic	57.44	56.02	2	68	0.86	0.43	
Correlational	55.81	55.19	1	66	0.74	0.39	

Univariate tests: $^{\mathbf{A}}$ P: $F_{1,70} = 5.09$, $P = 0.027$; C: $F_{1,70} = 1.81$, $P = 0.18$; $^{\mathbf{B}}$ P × P: $F_{1,66} = 5.17$, $P = 0.026$; C × C: $F_{1,66} = 1.66$, $P = 0.20$.

Table S5. Sequential model comparing the linear and nonlinear effects of protein (P) and carbohydrate (C) intake on protein carbonylation (PC) and total antioxidant capacity (TAC) across the sexes in *T. commodus* with and without DL-alpha-tocopherol supplementation. SS refers to sums of squares of the reduced (SS_r) and the complete (SS_c) models. DF quantifies the degrees of freedom and F is the test statistic.

	SS_R	SS_C	DF_1	DF_2	F	p Value
Supplemented PC						
Linear	73.09	67.04	2	72	3.25	**0.04** [A]
Quadratic	66.62	64.89	2	68	0.91	0.41
Correlational	64.87	64.86	1	66	0.01	0.92
Non-supplemented PC						
Linear	64.85	64.83	2	72	0.01	0.99
Quadratic	60.61	54.10	2	68	4.09	**0.02** [B]
Correlational	52.73	52.59	1	66	0.18	0.68
Supplemented TAC						
Linear	75.96	71.89	2	72	2.04	0.14
Quadratic	67.34	64.11	2	68	1.71	0.19
Correlational	63.90	62.89	1	66	1.06	0.31
Non-supplemented TAC						
Linear	72.28	71.30	2	72	0.49	0.61
Quadratic	66.63	66.01	2	68	0.32	0.73
Correlational	65.78	65.53	1	66	0.25	0.62

Univariate tests: [A] P: $F_{1,72} = 5.37$, $P = 0.023$; C: $F_{1,72} = 2.76$, $P = 0.10$; [B] P × P: $F_{1,68} = 5.58$, $P = 0.021$; C × C: $F_{1,68} = 0.73$, $P = 0.40$.

Table S6. Sequential model comparing the linear and nonlinear effects of protein (P) and carbohydrate (C) intake on lifespan (LS), daily reproductive effort (DRE), protein carbonylation (PC) and total antioxidant capacity (TAC) in female *T. commodus* with and without DL-alpha-tocopherol supplementation. DF quantifies the degrees of freedom, F the test statistic and ***θ*** the angle separating peaks for the traits being compared, with associated 95% confidence intervals.

	SS_R	SS_C	DF_1	DF_2	*F*	*p* Value	*θ* (95% CI)
Supplemented LS vs. PC							
Linear	65.85	64.99	2	73	0.48	0.62	38.71° (18.65°, 65.22°)
Quadratic	63.85	63.23	2	69	0.34	0.71	
Correlational	59.96	58.74	1	67	1.39	0.24	
Supplemented LS vs. TAC							
Linear	69.45	67.94	2	73	0.81	0.45	43.56° (22.67°, 67.09°)
Quadratic	67.64	65.26	2	69	1.26	0.29	
Correlational	62.60	60.78	1	67	2.01	0.16	
Supplemented DRE vs. PC							
Linear	76.22	69.49	2	73	3.53	**0.03** [A]	72.35° (43.33°, 97.76°)
Quadratic	67.95	62.04	2	69	3.29	**0.04** [B]	
Correlational	61.84	61.71	2	67	0.14	0.71	
Supplemented DRE vs. TAC							
Linear	79.81	72.44	2	73	3.71	**0.03** [C]	86.24° (46.22°, 117.60°)
Quadratic	71.46	64.45	2	69	3.75	**0.03** [D]	
Correlational	64.10	63.74	1	67	0.38	0.54	
Non-supplemented LS vs. PC							
Linear	56.34	53.96	2	72	1.59	0.21	37.24° (22.08°, 58.09°)
Quadratic	53.62	51.53	2	68	1.38	0.26	
Correlational	51.53	50.06	1	66	1.94	0.17	
Non-supplemented LS vs. TAC							
Linear	66.02	59.34	2	72	4.05	**0.02** [E]	81.20° (51.33°, 106.90°)
Quadratic	57.15	55.21	2	68	1.19	0.31	
Correlational	55.10	54.07	1	66	1.26	0.27	
Non-supplemented DRE vs. PC							
Linear	57.10	52.28	2	72	3.32	**0.04** [F]	76.39° (41.70°, 109.10°)
Quadratic	52.27	50.45	2	68	1.23	0.30	
Correlational	49.46	49.39	1	66	0.09	0.76	
Non-supplemented DRE vs. TAC							
Linear	60.16	57.66	2	72	1.56	0.22	94.70° (50.57°,138.50°)
Quadratic	57.03	54.12	2	68	1.83	0.17	
Correlational	53.43	53.41	1	66	0.02	0.88	

Univariate tests: [A] P: $F_{1,73} = 2.60$, $P = 0.11$; C: $F_{1,73} = 6.02$, $P = 0.017$; [B] P × P: $F_{1,69} = 4.61$, $P = 0.04$; C × C: $F_{1,69} = 0.14$, $P = 0.71$; [C] P: $F_{1,73} = 1.65$, $P = 0.20$; C: $F_{1,73} = 7.04$, $P = 0.01$; [D] P × P: $F_{1,69} = 5.92$, $P = 0.018$; C × C: $F_{1,69} = 1.31$, $P = 0.26$; [E] P: $F_{1,72} = 0.11$, $P = 0.74$; C: $F_{1,72} = 5.63$, $P = 0.02$; [F] P: $F_{1,72} = 4.56$, $P = 0.036$; C: $F_{1,72} = 0.66$, $P = 0.42$.

Table S7. Sequential model comparing the linear and nonlinear effects of protein (P) and carbohydrate (C) intake on lifespan (LS), daily reproductive effort (DRE), protein carbonylation (PC) and total antioxidant capacity (TAC) in male *T. commodus* with and without DL-alpha-tocopherol supplementation. DF quantifies the degrees of freedom, F the test statistic and ***θ*** the angle separating peaks for the traits being compared, with associated 95% confidence intervals.

	SS_R	SS_C	DF_1	DF_2	F	***p* Value**	***θ* (95% CI)**
Supplemented LS vs. PC							
Linear	64.16	63.91	2	71	0.14	0.87	25.52° (11.50°, 48.09°)
Quadratic	61.86	61.80	2	67	0.03	0.97	
Correlational	61.56	61.43	1	65	0.14	0.71	
Supplemented LS vs. TAC							
Linear	72.05	65.83	2	71	3.35	**0.04** [A]	92.18° (58.42°, 129.40°)
Quadratic	60.00	59.00	2	67	0.57	0.57	
Correlational	57.76	57.44	1	65	0.36	0.55	
Supplemented DRE vs. PC							
Linear	76.76	76.17	2	71	0.27	0.76	42.52° (20.41°, 85.30°)
Quadratic	75.28	74.53	2	67	0.34	0.72	
Correlational	74.52	74.51	1	65	0.01	0.93	
Supplemented DRE vs. TAC							
Linear	80.62	78.09	2	71	1.15	0.32	103.50° (66.10°, 144.80°)
Quadratic	75.20	71.73	2	67	1.62	0.21	
Correlational	71.22	70.52	1	65	0.65	0.42	
Non-supplemented LS vs. PC							
Linear	67.62	61.65	2	72	3.48	**0.04** [B]	73.10° (38.95°, 103.60°)
Quadratic	56.96	50.34	2	68	4.47	**0.01** [C]	
Correlational	50.32	48.14	1	66	2.99	0.09	
Non-supplemented LS vs. TAC							
Linear	72.31	62.72	2	72	5.50	**0.01** [D]	116.00° (85.77°, 153.60°)
Quadratic	62.16	58.64	2	68	2.04	0.14	
Correlational	57.57	57.06	1	66	0.59	0.45	
Non-supplemented DRE vs. PC							
Linear	65.24	63.32	2	70	1.06	0.35	79.57° (41.79°, 119.60°)
Quadratic	55.44	52.37	2	66	1.93	0.15	
Correlational	52.29	49.66	1	64	3.39	0.07	
Non-supplemented DRE vs. TAC							
Linear	66.13	64.40	2	70	0.94	0.40	104.60° (62.61°, 149.30°)
Quadratic	62.29	60.58	2	66	0.93	0.40	
Correlational	59.34	58.58	1	64	0.83	0.37	

Univariate tests: [A] P: $F_{1,71} = 0.36$, $P = 0.55$; C: $F_{1,71} = 4.81$, $P = 0.031$; [B] P: $F_{1,72} = 6.67$, $P = 0.012$; C: $F_{1,72} = 4.38$, $P = 0.040$; [C] P × P: $F_{1,68} = 8.72$, $P = 0.004$; C × C: $F_{1,68} = 0.14$, $P = 0.71$; [D] P: $F_{1,72} = 7.34$, $P = 0.008$; C: $F_{1,72} = 10.29$, $P = 0.002$.

Table S8. Results of some studies examining how dietary manipulation affects oxidative protection (antioxidant levels), ROS production, or oxidative damage. This is not intended to be a comprehensive list but simply to illustrate the complex associations between dietary manipulation, oxidative damage and protection in a range of taxa. **Abbreviations**: **Sex**: NA—not appropriate, F = female, M = male. **Dietary manipulation**: AAR—amino acid restriction, DR—total food or energy restriction, GlucR—Glucose Restriction, PR—protein restriction, MetR—methionine restriction. **Results**: CAT—antioxidant catalase, GSH—antioxidant glutathione, GPX-antioxidant glutathione peroxidase, mtDNA—mitochondrial DNA, nDNA—nuclear DNA, SOD-antioxidant superoxide dismutase. **Symbols**: ↑—increased, "−"—no significant difference, ↓—reduced. All comparisons are relative to control animals e.g., DR increases damage relative to controls. If two symbols are provided, results differed across tissue types or over time.

Sex	Dietary Manipulation	Results	Reference
Caernorrhabditis elegans			
NA	Axenic media	**Protection** SOD ↑; CAT ↑	[5,6]
NA	DR	**Protection** SOD ↑; CAT ↑/− *: **Resistance to OS** ↓/−	[7]
NA	GlucR †	**Protection** CAT ↑; SOD −; GPX − : **ROS Production** ↑: **Resistance to OS** ↑	[8]
Drosophila melanogaster			
F	DR	**Damage** lipid delayed	[9]
F	DR	**Resistance to OS** ↓	[10]
**Mice**			
M	CR	**Damage** DNA ↓/− ‡	[11]
M	DR	**Damage** protein ↓: **Protection** SOD −; CAT ↑; GPX −: **ROS Production** ↓	[12]
M	DR	**Damage** protein ↓	[13]
M	DR	**Damage** lipid ↓: **ROS Production** ↓	[14]
**Rats**			
M	DR	**Protection** CAT ↑; GPX ↑; GSH −; SOD −: **ROS Production** ↓/−	[15]
M	PR & DR	**Damage** protein ↓	[16]
M	DR	**Damage** DNA ↓/−	[17]
M	DR	**Damage** protein ↓/−: **Protection** SOD ↓; GPX ↓; CAT ↓	[18]
M	DR	**Damage** mDNA ↓; nDNA −	[19]
M	DR	**Protection** CAT −; SOD −: **Damage** lipid ↓	[20]
M	DR	**Damage** nDNA −; mtDNA ↓: **ROS Production** ↓/−	[21]
M	DR	**Damage** protein ↓	[22]
M	DR	**Damage** DNA ↓: **ROS Production** ↓	[23]
M	MetR	**Damage** DNA ↓; lipid ↓; protein ↓: **ROS Production** ↓	[24]
M	AAR	**Damage** DNA −; lipid ↓, protein ↓: **ROS Production** −	[25]
M	DR	**Damage** mtDNA −, lipid ↓; protein ↓: **ROS Production** ↓	[26]
M	DR	**Damage** DNA ↓; protein ↓: **ROS Production** ↓	[27]
M	DR (+/−extra fibre)	**Damage** lipid ↓ **Protection** SOD ↑; CAT ↑; GSH ↑	[28]
M	DR (short/long term)	**Damage** short term −; long term mtDNA ↓; nDNA −: **ROS Production** short term −; long term ↓	[29]
M F	DR	**Damage** protein ↓	[30]
**Rhesus monkeys**			
M	DR	**Damage** protein ↓	[31]
Saccharomyces cerevisiae			
NA	DR	**Protection** CAT ↑; SOD ↑; GPX ↑: **Resistance to OS** ↓: **ROS Production** ↑	[32]

* *Result depended on strain studied.* † Glucose Restriction achieved via exposure to a chemical inhibitor of glycolysis. ‡ Results tissue specific.

References

1. Maklakov, A.A.; Simpson, S.J.; Zajitschek, F.; Hall, M.D.; Dessmann, J.; Clissold, F.; Raubenheimer, D.; Bonduriansky, R.; Brooks, R.C. Sex-specific fitness effects of nutrient intake on reproduction and lifespan. *Curr. Biol.* **2008**, *18*, 1062–1066.
2. Draper, N.R.; John, J.A. Response-surface designs for quantitative and qualitative variables. *Technometrics* **1988**, *30*, 423–428.
3. Chenoweth, S.F.; Blows, M.W. Contrasting mutual sexual selection on homologous signal traits in *Drosophila serrata*. *Am. Nat.* **2005**, *165*, 281–289.
4. Bowerman, B.L.; O'Connell, R.T. *Linear Statistical Models: An Applied Approach*; PWS-Kent Publishing: Boston, MA, USA, 1990.
5. Houthoofd, K.; Braeckman, B.P.; Lenaerts, I.; Brys, K.; de Vreese, A.; van Eygen, S.; Vanfleteren, J.R. Axenic growth up-regulates mass-specific metabolic rate, stress resistance, and extends life span in *Caenorhabditis elegans*. *Exp. Gerontol.* **2002**, *37*, 1371–1378.
6. Houthoofd, K.; Braeckman, B.P.; Johnson, T.E.; Vanfleteren, J.R. Life extension via dietary restriction is independent of the Ins/IGF-1 signalling pathway in *Caenorhabditis elegans*. *Exp. Gerontol.* **2003**, *38*, 947–954.
7. Houthoofd, K.; Braeckman, B.P.; Lenaerts, I.; Brys, K.; de Vreese, A.; van Eygen, S.; Vanfleteren, J.R. No reduction of metabolic rate in food restricted *Caenorhabditis elegans*. *Exp. Gerontol.* **2002**, *37*, 1359–1369.
8. Schulz, T.J.; Zarse, K.; Voigt, A.; Urban, N.; Birringer, M.; Ristow, M. Glucose restriction extends *Caenorhabditis elegans* life span by inducing mitochondrial respiration and increasing oxidative stress. *Cell Metab.* **2007**, *6*, 280–293.
9. Zheng, J.; Mutcherson, R., II; Helfand, S.L. Calorie restriction delays lipid oxidative damage in Drosophila melanogaster. *Aging Cell* **2005**, *4*, 209–216.
10. Burger, J.; Hwangbo, D.S.; Corby-Harris, V.; Promislow, D.E.L. The functional costs and benefits of dietary restriction in Drosophila. *Aging Cell* **2007**, *6*, 63–71.
11. Sohal, R.S.; Agarwal, S.; Candas, M.; Forster, M.J.; Lal, H. Effect of age and caloric restriction on DNA oxidative damage in different tissues of C57BL/6 mice. *Mech. Ageing Dev.* **1994**, *76*, 215–224.
12. Sohal, R.S.; Ku, H.H.; Agarwal, S.; Forster, M.J.; Lal, H. Oxidative damage, mitochondrial oxidant generation and antioxidant defenses during aging and in response to food restriction in the mouse. *Mech. Ageing Dev.* **1994**, *74*, 121–133.
13. Dubey, A.; Forster, M.J.; Lal, H.; Sohal, R.S. Effect of age and caloric intake on protein oxidation in different brain regions and on behavioral functions of the mouse. *Arch. Biochem. Biophys.* **1996**, *333*, 189–197.
14. Guo, Z.M.; Mitchell-Raymundo, F.; Yang, H.; Ikeno, Y.; Nelson, J.; Diaz, V.; Richardson, A.; Reddick, R. Dietary restriction reduces atherosclerosis and oxidative stress in the aorta of apolipoprotein E-deficient mice. *Mech. Ageing Dev.* **2002**, *123*, 1121–1131.
15. Kim, J.; McCarter, R.; Yu, B. Influence of age, exercise, and dietary restriction on oxidative stress in rats. *Aging* **1996**, *8*, 123–129.
16. Youngman, L.D.; Park, J.Y.; Ames, B.N. Protein oxidation associated with aging is reduced by dietary restriction of protein or calories. *Proc. Natl. Acad. Sci. USA* **1992**, *89*, 9112–9116.

17. Kaneko, T.; Tahara, S.; Matsuo, M. Retarding effect of dietary restriction on the accumulation of 8-hydroxy-2′-deoxyguanosine in organs of Fischer 344 rats during aging. *Free Radic. Biol. Med.* **1997**, *23*, 76–81.
18. Radák, Z.; Takahashi, R.; Kumiyama, A.; Nakamoto, H.; Ohno, H.; Ookawara, T.; Goto, S. Effect of aging and late onset dietary restriction on antioxidant enzymes and proteasome activities, and protein carbonylation of rat skeletal muscle and tendon. *Exp. Gerontol.* **2002**, *37*, 1423–1430.
19. Chung, M.H.; Kasai, H.; Nishimura, S.; Yu, B.P. Protection of DNA damage by dietary restriction. *Free Radic. Biol. Med.* **1992**, *12*, 523–525.
20. Jeon, T.I.; Lim, B.O.; Yu, B.P.; Lim, Y.; Jeon, E.J.; Park, D.K. Effect of dietary restriction on age-related increase of liver susceptibility to peroxidation in rats. *Lipids* **2001**, *36*, 589–593.
21. López-Torres, M.; Gredilla, R.; Sanz, A.; Barja, G. Influence of aging and long-term caloric restriction on oxygen radical generation and oxidative DNA damage in rat liver mitochondria. *Free Radic. Biol. Med.* **2002**, *32*, 882–889.
22. Pamplona, R.; Portero-Otín, M.; Requena, J.; Gredilla, R.; Barja, G. Oxidative, glycoxidative and lipoxidative damage to rat heart mitochondrial proteins is lower after 4 months of caloric restriction than in age-matched controls. *Mech. Ageing and Dev.* **2002**, *123*, 1437–1446.
23. Sanz, A.; Caro, P.; Barja, G. Protein restriction without strong caloric restriction decreases mitochondrial oxygen radical production and oxidative DNA damage in rat liver. *J. Bioenerg. Biomembr.* **2004**, *36*, 545–552.
24. Caro, P.; Gómez, J.; López-Torres, M.; Sánchez, I.; Naudí, A.; Jove, M.; Pamplona, R.; Barja, G. Forty percent and eighty percent methionine restriction decrease mitochondrial ROS generation and oxidative stress in rat liver. *Biogerontology* **2008**, *9*, 183–196.
25. Caro, P.; Gomez, J.; Sanchez, I.; Garcia, R.; López-Torres, M.; Naudí, A.; Portero-Otin, M.; Pamplona, R.; Barja, G. Effect of 40% restriction of dietary amino acids (except methionine) on mitochondrial oxidative stress and biogenesis, AIF and SIRT1 in rat liver. *Biogerontology* **2009**, *10*, 579–592.
26. Gómez, J.; Caro, P.; Naudí, A.; Portero-Otin, M.; Pamplona, R.; Barja, G. Effect of 8.5% and 25% caloric restriction on mitochondrial free radical production and oxidative stress in rat liver. *Biogerontology* **2007**, *8*, 555–566.
27. Ayala, V.; Naudí, A.; Sanz, A.; Caro, P.; Portero-Otin, M.; Barja, G.; Pamplona, R. Dietary protein restriction decreases oxidative protein damage, peroxidizability index, and mitochondrial complex I content in rat liver. *J. Gerontol. Ser. Biol. Sci. Med. Sciences* **2007**, *62*, 352–360.
28. Diniz, Y.S.; Cicogna, A.C.; Padovani, C.R.; Silva, M.D.P.; Faine, L.A.; Galhardi, C.M.; Rodrigues, H.G.; Novelli, E.L.B. Dietary restriction and fibre supplementation: Oxidative stress and metabolic shifting for cardiac health. *Can. J. Physiol. Pharmacol.* **2003**, *81*, 1042–1048.
29. Gredilla, R.; Sanz, A.; Lopez-Torres, M.; Barja, G. Caloric restriction decreases mitochondrial free radical generation at complex I and lowers oxidative damage to mitochondrial DNA in the rat heart. *FASEB J.* **2001**, *15*, 1589.

30. Aksenova, M.V.; Aksenov, M.Y.; Carney, J.M.; Butterfield, D.A. Protein oxidation and enzyme activity decline in old brown Norway rats are reduced by dietary restriction. *Mech. Ageing Dev.* **1998**, *100*, 157–168.
31. Zainal, T.A.; Oberly, T.D.; Allison, D.B.; Szweda, L.I.; Weindruch, R. Caloric restriction of rhesus monkeys lowers oxidative damage in skeletal muscle. *FASEB J.* **2000**, *14*, 1825–1836.
32. Agarwal, S.; Sharma, S.; Agrawal, V.; Roy, N. Caloric restriction augments ROS defense in *S. cerevisiae*, by a Sir2p independent mechanism. *Free Radic. Res.* **2005**, *39*, 55–62.

© 2015 by the authors; licensee MDPI, Basel, Switzerland. This article is an open access article distributed under the terms and conditions of the Creative Commons Attribution license (http://creativecommons.org/licenses/by/4.0/).
